# Progressive Gingival Digital Workflow with Chairside Abutment Customization for Cement-Retained Implant Restorations: A Pilot Comparative Clinical Study of Two Treatment Concepts

**DOI:** 10.3390/dj14080484

**Published:** 2026-08-05

**Authors:** Dragos Epistatu, Maria Teodora Epistatu, Malina Elena Meila, Valentin Daniel Sirbu, Andreea Mihaela Custura, Ioan Sirbu

**Affiliations:** 1 Department of Dental Radiology, Faculty of Dentistry, “Carol Davila” University of Medicine and Pharmacy, 020021 Bucharest, Romania; 2 Department of Oral Implantology, Faculty of Dentistry, “Carol Davila” University of Medicine and Pharmacy, 020021 Bucharest, Romania; 3 Department of Dental Prosthetic Technology, Faculty of Dentistry, “Carol Davila” University of Medicine and Pharmacy, 020021 Bucharest, Romania

**Keywords:** progressive gingival workflow, digital implant workflow, chairside customized abutments, peri-implant tissue conditioning, emergence profile management, cement-retained implant restorations, prosthetic protocol

## Abstract

**Background**: Digital workflows in implant prosthodontics continue to evolve, yet standardized protocols integrating clinician-driven abutment customization and peri-implant soft-tissue conditioning remain limited. This pilot study aimed to present and evaluate a progressive gingival digital workflow for cement-retained implant-supported restorations based on chairside adjustment of prefabricated abutments according to gingival maturation. **Methods**: A retrospective comparative study included 39 patients rehabilitated with 161 implants and followed for 24–48 months. The experimental group comprised 87 bone-level implants restored using a progressive digital workflow involving staged healing, chairside abutment customization, provisional tissue conditioning, and digital fabrication of definitive restorations. Outcomes were compared with 74 tissue-level implants restored using a conventional protocol with healing abutments and prefabricated prosthetic abutments. Clinical and radiographic outcomes included plaque index, bleeding on probing (BOP), peri-implant sulcus depth, marginal bone resorption, complications, and patient satisfaction. **Results**: Both groups demonstrated favorable clinical outcomes, with no significant differences in plaque index, gingival condition, BOP (28.74% vs. 21.62%), sulcus dimensions, or marginal bone resorption (11.49% vs. 12.16%) (*p* > 0.05). Logistic regression identified plaque accumulation, vestibular sulcus depth, and implant duration as predictors of BOP, whereas implant type was not associated with BOP or bone resorption. Patient satisfaction, assessed using a visual analog scale, was uniformly high. Most patients reported maximum scores across all domains, with only minor esthetic concerns reported by a few participants. No major prosthetic complications or screw loosening occurred during follow-up. **Conclusions**: The proposed workflow demonstrated favorable clinical performance and appears to be a feasible restorative approach. However, because implant design and restorative workflow differed simultaneously, findings should be interpreted cautiously. Larger prospective studies are needed to confirm these results.

## 1. Introduction

In contemporary practice, implant-supported prosthetic rehabilitation increasingly relies on digital workflows, although conventional methods have not been completely abandoned [1]. Technological advances in recent years have improved intraoral scanning, and the use of laboratory software enables the high-performance design of prosthetic restorations [2,3,4,5,6].

Several digital and hybrid workflows have been described in the literature [7,8,9,10,11,12,13,14]. At the same time, the relative advantages of cement-retained and screw-retained restorations remain debated, with conflicting evidence regarding esthetic, biological, and mechanical outcomes [15,16,17,18,19,20]. Some studies associate cement-retained restorations with improved esthetic integration and passive fit, whereas others emphasize the retrievability and maintenance advantages of screw-retained designs [15,16]. Reported differences regarding fracture resistance, technical complications, and peri-implant inflammatory parameters also remain inconsistent across the literature [21,22,23].

In addition to biological and technical differences, these methods may also involve different restorative strategies and clinical workflows, which could influence treatment planning, prosthetic management, and overall clinical predictability [4,6,24]. Workflows that primarily rely on laboratory-based customization may add more sources of biological or technical variability and complicate treatment. Protocols that maintain soft tissue stability while streamlining abutment management may therefore be clinically relevant [1,4]. In this context, simplified clinician-centered workflows that integrate digital technology without increasing procedural complexity may represent a relevant direction for implant-supported rehabilitation.

The development of a stable peri-implant emergence profile represents a critical step in implant-supported rehabilitation, particularly in esthetic areas [25,26,27]. Soft tissue architecture is influenced not only by implant position and tissue biotype, but also by the shape and timing of abutment and restoration modifications during the healing phase [27,28,29]. Repeated disconnection and replacement of healing abutments or prosthetic components have been associated with disturbances of the peri-implant mucosal seal and alterations in soft tissue stability [30,31,32]. Consequently, restorative concepts that promote gradual tissue conditioning while minimizing unnecessary component manipulation may offer biological advantages and facilitate emergence profile development [27,28,29].

Since peri-implant soft tissue care and emergence profile development are important factors in long-term biological and prosthetic success, they have received special attention in recent years [33,34]. Nevertheless, standardized digital protocols integrating staged soft-tissue conditioning, emergence profile management, and clinician-driven customization of prefabricated abutments remain insufficiently documented, particularly protocols combining staged peri-implant soft tissue conditioning with clinician-driven customization of prefabricated abutments [1,35,36].

Building upon these considerations, we developed a customized progressive gingival digital workflow based on staged peri-implant soft tissue conditioning and clinician-guided customization of prefabricated abutments according to the progressive maturation of peri-implant tissues. The workflow integrates contemporary digital restorative procedures while aiming to preserve soft tissue stability throughout the restorative phase.

The aim of this pilot comparative study was to describe the proposed treatment concept and explore its clinical performance within an integrated restorative strategy based on bone-level implants, compared with an established tissue-level treatment concept.

The null hypothesis was that no significant differences would exist between the two protocols regarding the evaluated clinical and radiographic outcomes.

## 2. Materials and Methods

### 2.1. Study Design

**Figure 1:**
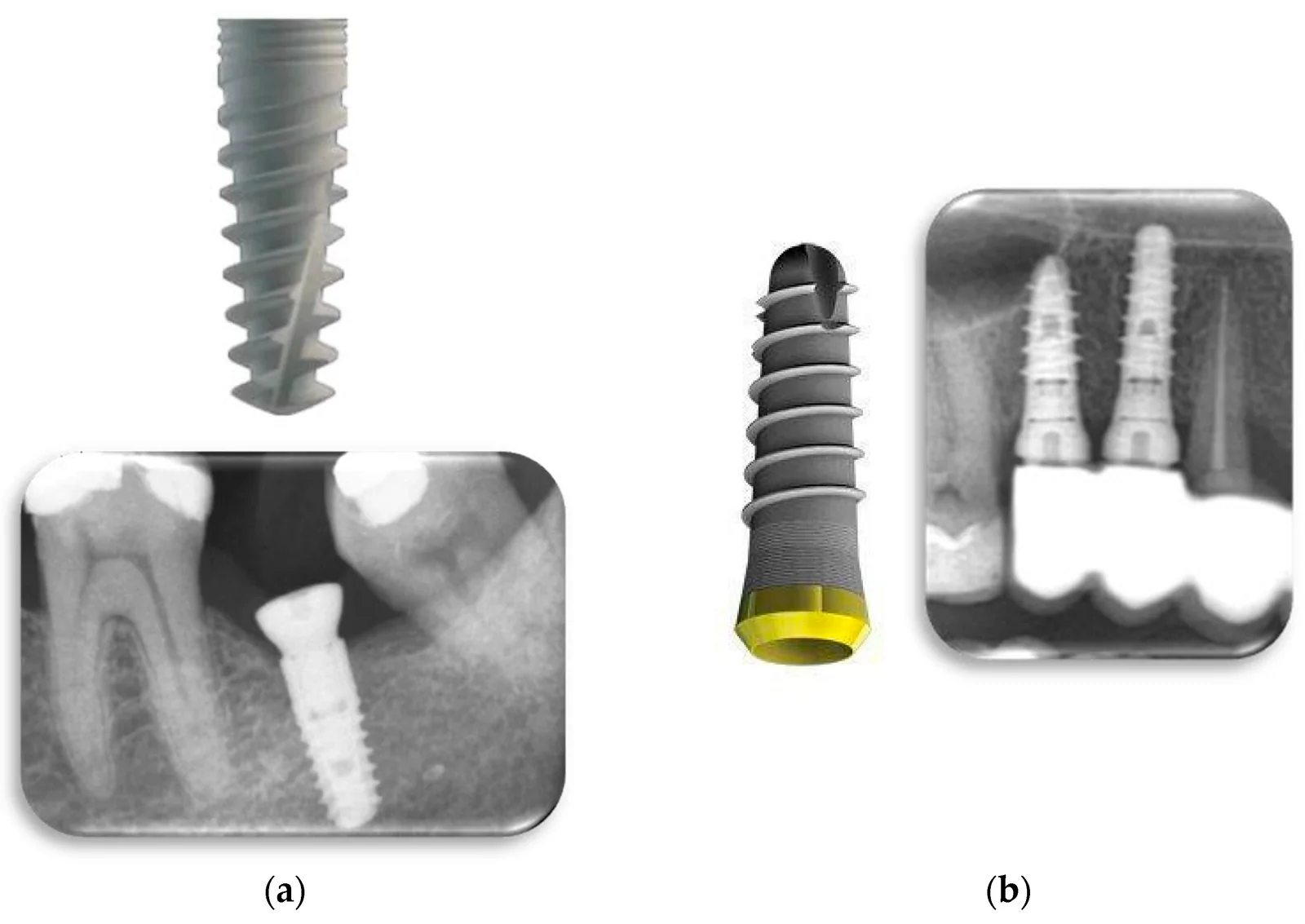
Implant systems included in the study: (**a**) Nova bone-level implant, characterized by a conical internal connection and crestal-level positioning [37], and (**b**) Exacta tissue-level implant, characterized by a transmucosal polished collar and tissue-level prosthetic platform [38].

A retrospective comparative observational clinical and radiographic pilot study was conducted on patients treated in a private clinical practice and followed for 24–48 months under two different prosthetic protocols. Patients were consecutively selected from the clinical records according to predefined eligibility criteria. No additional patient selection was performed beyond the predefined eligibility criteria. The experimental group received Nova bone-level implants (Nova^®^ Implants Medical Devices & Development Ltd., Or Yehuda, Israel) (Figure 1) with diameters of 3.75 and 4.2 mm and lengths of 8, 10, 11.5, and 13 mm and fixed cement-retained restorations using a standardized progressive gingival digital workflow. The control group received Exacta tissue-level implants (Exacta^®^ Biaggini Medical, Arcola/La Spezia, Italy) (Figure 1) with diameters of 4 and 4.3 mm and lengths of 9, 11, and 13 mm, restored under an established prosthetic protocol. Treatment allocation was based on implant system selection and clinical preference during the study period; no randomization was performed.

Owing to its exploratory nature, this investigation was designed as a comparative pilot study. The comparison evaluated the clinical performance of two integrated restorative protocols as applied in practice. The objective was not to isolate the effect of implant design, but rather to evaluate two complete treatment concepts combining different implant configurations and restorative workflows under routine clinical conditions. Although the restorative workflows differed in implant configuration and abutment management, both protocols followed conventional delayed-loading principles and aimed to achieve stable implant-supported rehabilitation under routine clinical conditions.

This retrospective study was conducted in accordance with the ethical principles of the Declaration of Helsinki (1975, revised in 2013). The clinical protocol received Ethics Committee approval from the Department of Oral Implantology and Dental Radiology, Faculty of Dentistry, “Carol Davila” University of Medicine and Pharmacy, Bucharest (Approval No. 04, 13 September 2021). Subsequently, the Ethics Committee reviewed and approved the final stage of the research, including the collection and analysis of clinical and radiographic data (21 July 2025). Written informed consent was obtained from all participants for treatment and for the use of anonymized clinical and radiographic data for research purposes.

In the experimental group, bone-level implants were placed at the level of the cortical crest and restored according to the progressive gingival digital workflow described below, which was structured according to progressive stages of gingival maturation. The implant–abutment connection of the experimental implants was conical with an internal hex configuration.

**Figure 2:**
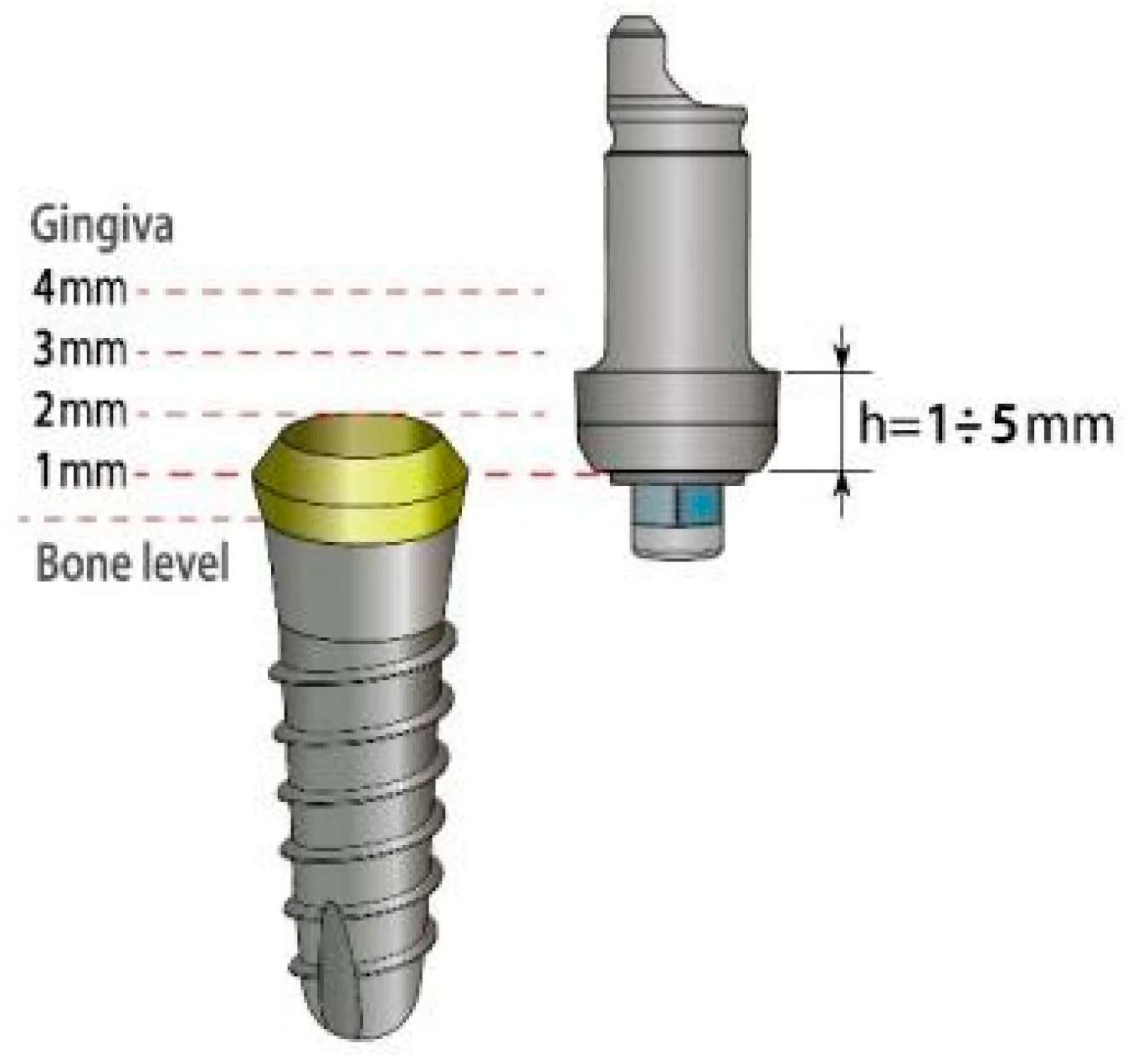
Schematic representation of the tissue-level implant restorative concept used in the control group, illustrating transmucosal implant positioning, prosthetic abutment shoulder heights (h), and their relationship to the gingival margin and alveolar bone crest.

In contrast, for the tissue-level implants (control group), the polished neck of the implants traversed the thickness of the gingival tissue at the time of insertion, corresponding to a transmucosal height of 1 mm. The implants were inserted according to the manufacturer’s surgical protocol, with the transmucosal collar remaining above the crestal bone level. In the control group, shoulder height selection was adapted according to soft tissue thickness, being positioned at gingival level in posterior areas and 1–2 mm subgingivally within the esthetic zone. Healing abutments were placed at the moment of implant insertion. Accordingly, prosthetic procedures were performed at least 1 mm coronal to the alveolar bone crest. In the control group, prefabricated abutments with shoulder heights of 1–5 mm were selected according to the peri-implant soft tissue profile (Figure 2).

### 2.2. Inclusion/Exclusion Criteria

Inclusion criteria

• Patients treated according to one of the two predefined prosthetic protocols included in the study;
• Submerged implant placement without healing abutments (for experimental group only) and with healing abutments (for control group);
• Patients restored with fixed cement-retained implant-supported prostheses;
• Conventional delayed prosthetic protocol (4–6 months);
• Written informed consent for inclusion in the retrospective analysis and use of anonymized clinical and radiographic data.

Exclusion criteria

• Immediate-loading cases, due to a substantially different restorative sequence;
• Age under 18 or lack of legal capacity;
• Pregnancy;
• Presence of untreated periodontal disease;
• Alcohol or drug addiction;
• Bisphosphonate treatment;
• Head and neck radiation therapy;
• Severe bruxism;
• Uncontrolled systemic infectious diseases (B/C hepatitis or AIDS);
• Severe xerostomia;
• Implant failure or bone resorption before loading the implants;
• Mobile dentures as antagonists;
• Uncontrolled metabolic or endocrine disorders;
• History of severe periodontitis;
• Heavy smoking (>10 cigarettes/day);
• Uncontrolled autoimmune disorders;
• Long-term systemic corticosteroid therapy.

### 2.3. Outcome Assessment

The primary outcome measures included marginal bone loss and bleeding on probing. The secondary outcome measures included plaque index, peri-implant sulcus depth (vestibular and oral), gingival condition, recorded complications, and patient satisfaction, which was assessed at the time of the final follow-up visit using a visual analog scale (VAS). Patients rated esthetics, comfort, chewing ability, speech, stability, and overall treatment outcome on a scale from 0 (completely dissatisfied) to 10 (completely satisfied). Demographic and baseline variables including age, sex, implant position, and gingival phenotype were also recorded.

Plaque accumulation, probing depth, bleeding on probing, and gingival condition were clinically assessed at follow-up using standardized clinical examination procedures. Prosthetic and biological complications were recorded during follow-up. Dental plaque was assessed using the Silness and Löe Plaque Index. The surfaces of the selected teeth were examined and scored from 0 to 3 according to the amount of plaque present. The plaque index for each participant was calculated as the mean score of all examined surfaces. Bleeding on probing (BOP) was assessed using a standardized periodontal probe. The probe was gently inserted into the buccal and lingual peri-implant sulcus at six sites per implant. Bleeding occurring within 15 s after probing was recorded as present (score = 1), whereas the absence of bleeding was recorded as absent (score = 0).

Marginal bone loss was evaluated on standardized digital periapical radiographs obtained using the parallel technique. The vertical distance from the implant platform to the first bone-to-implant contact was measured on both the mesial and distal aspects of each implant. Measurements were recorded in millimeters, and the mean value of the mesial and distal measurements was used for statistical analysis.

**Figure 3:**
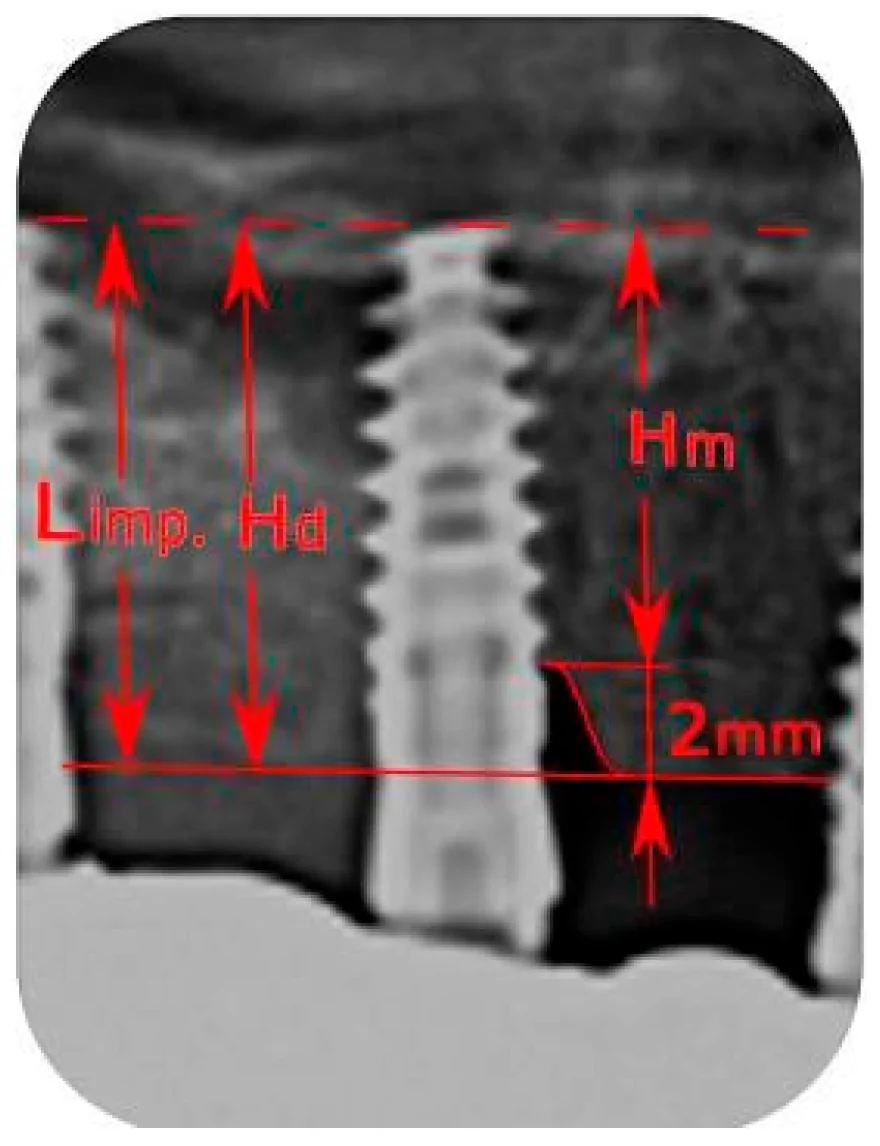
Representative standardized periapical radiograph used for marginal bone-level assessment around a dental implant. Implant length (Limp) and mesial and distal bone height measurements (Hm and Hd) were used to calculate marginal bone resorption relative to the implant–abutment reference level. Measurements were performed on calibrated digital radiographs obtained using the paralleling technique.

Radiographic assessment of marginal bone levels was performed at two predefined time points: T1, corresponding to the time of prosthetic loading (baseline radiographic evaluation), and T2, corresponding to the most recent follow-up examination performed between 24 and 48 months after loading. Marginal bone resorption was calculated as the difference between T1 and T2 measurements. Only panoramic radiographs with appropriate patient positioning and image quality were accepted, including correct midline centering and Frankfurt horizontal plane orientation within ±5° of the true horizontal plane [39]. In addition, to improve radiographic standardization, intraoral periapical radiographs using the parallel cone technique for all implants were used for the assessment and measurement of marginal bone resorption (Figure 3). Radiographic measurements were performed independently by three observers with experience in radiology and implant-prosthetic treatments. The results were subsequently compared. Measurements were jointly reviewed in cases of discrepancy to minimize observer-related variability. Standardized digital periapical radiographs were obtained using the paralleling technique with a Rinn XCP positioning system (Dentsply Sirona, Charlotte, NC, USA) and photostimulable phosphor sensors (Carestream Dental LLC, Atlanta, GA, USA) [40].

Known implant length was used as a radiographic calibration reference to verify image isometry and improve measurement accuracy.

Marginal bone levels were measured mesially and distally for each implant on standardized periapical radiographs, and the arithmetic mean was calculated.

### 2.4. Progressive Gingival Digital Workflow

The workflow consisted of five sequential clinical stages summarized in Table 1. Standard prefabricated prosthetic abutments were used in combination with PMMA provisional and zirconia definitive restorations.

All restorative procedures were performed by the same clinician.

#### 2.4.1. Implant Uncovering and Tissue Conditioning

**Figure 4:**
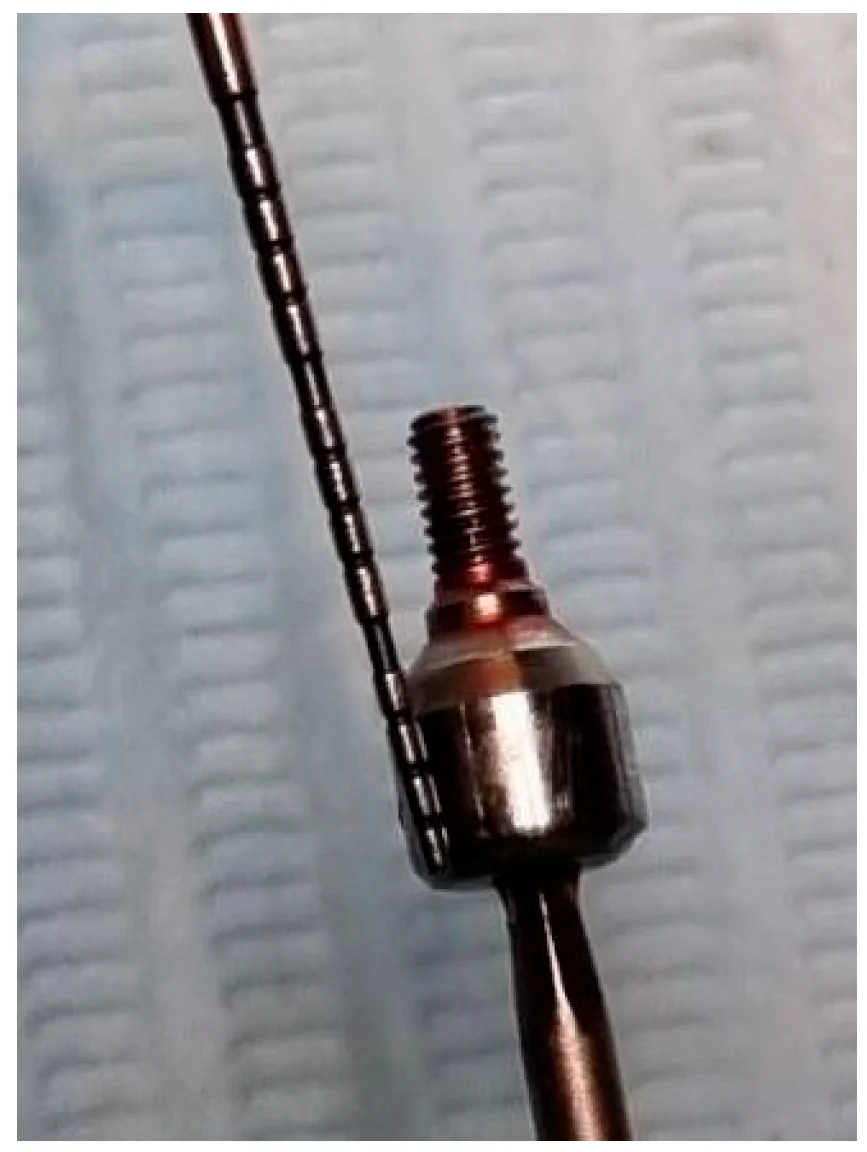
Healing abutment (3 mm).

Surgical uncovering of the implants was performed at approximately 3–4 months in the mandible and 5–6 months in the maxilla (first prosthetic appointment). The uncovering appointment was critical for gingival management. The incision was made along the mid-crestal line or slightly more orally to advance as much keratinized tissue as possible to the buccal aspect of the healing abutments placed at that certain moment. Gingival management also included assessment of tissue thickness, which should not exceed 3 mm. In cases of thick gingiva, either a connective tissue excision (wedge-shaped) was performed, or the excess gingival tissue was removed with a bur/electrocautery/or laser after placement of the healing abutments [41]. The 3 mm target thickness was adopted as a practical clinical objective within the present protocol and should not be interpreted as a universally applicable biological threshold. Healing abutments had a maximum height of 3 mm (Figure 4) to allow evaluation of gingival height after suturing. Implant uncovering was followed by an assessment of implant stability using a Periotest M device (Medizintechnik Gulden, Modautal, Germany).

**Figure 5:**
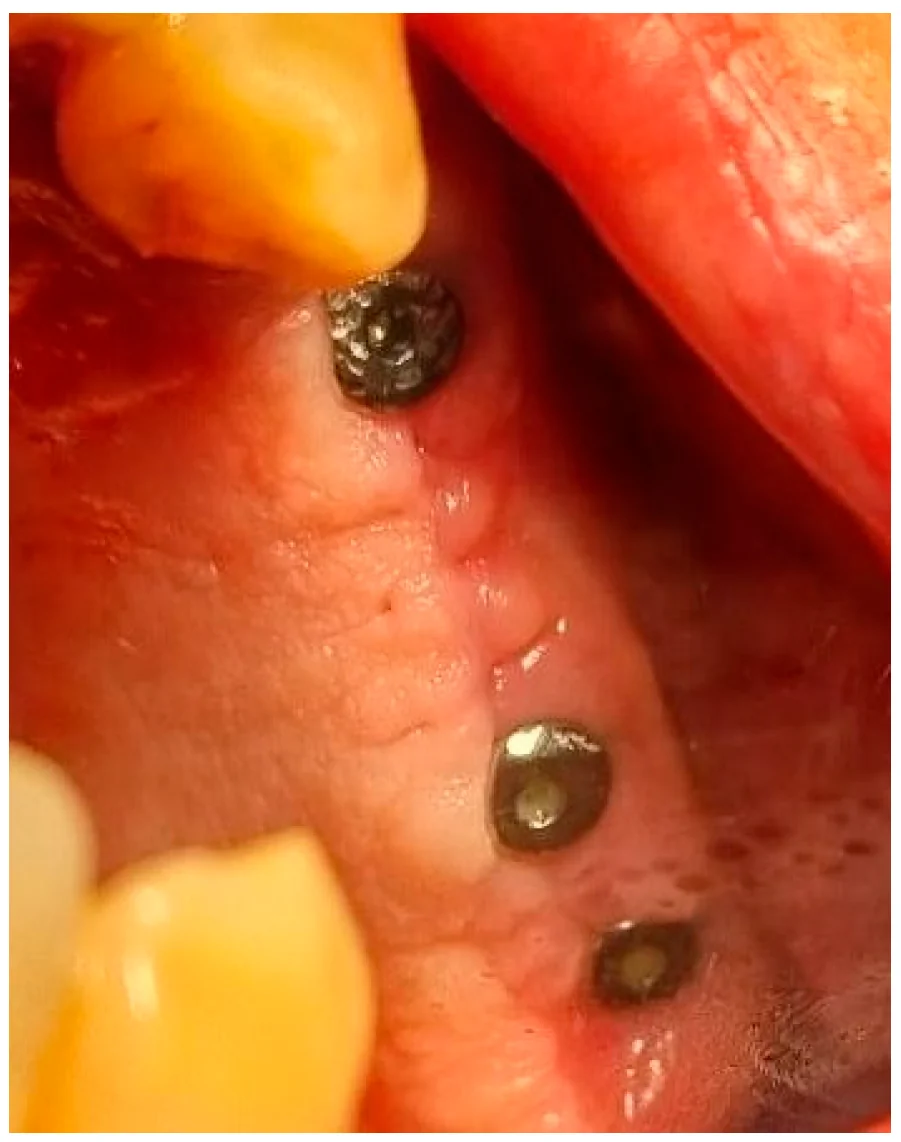
Soft tissue appearance 2–3 weeks after implant exposure.

After placement of the healing abutments, the site was sutured with interrupted stitches. After this appointment, we allowed a two-week period for gingival healing (Figure 5).

#### 2.4.2. Chairside Abutment Customization

**Figure 6:**
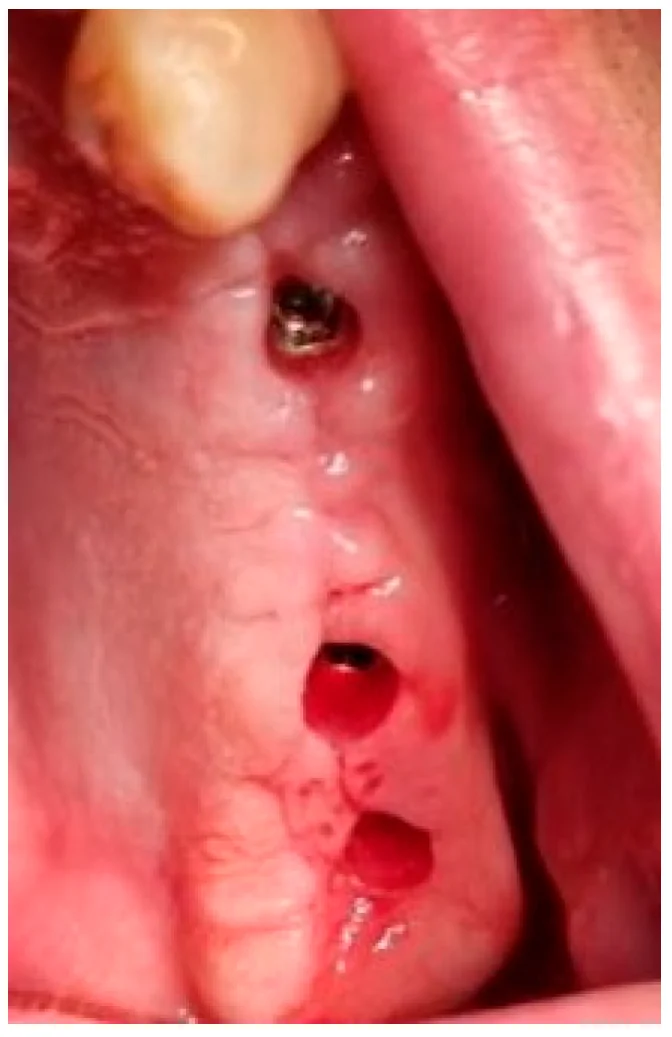
Gingival shaping after removal of the healing abutments; the thickness of the gingiva may vary from one area to another.

**Figure 7:**
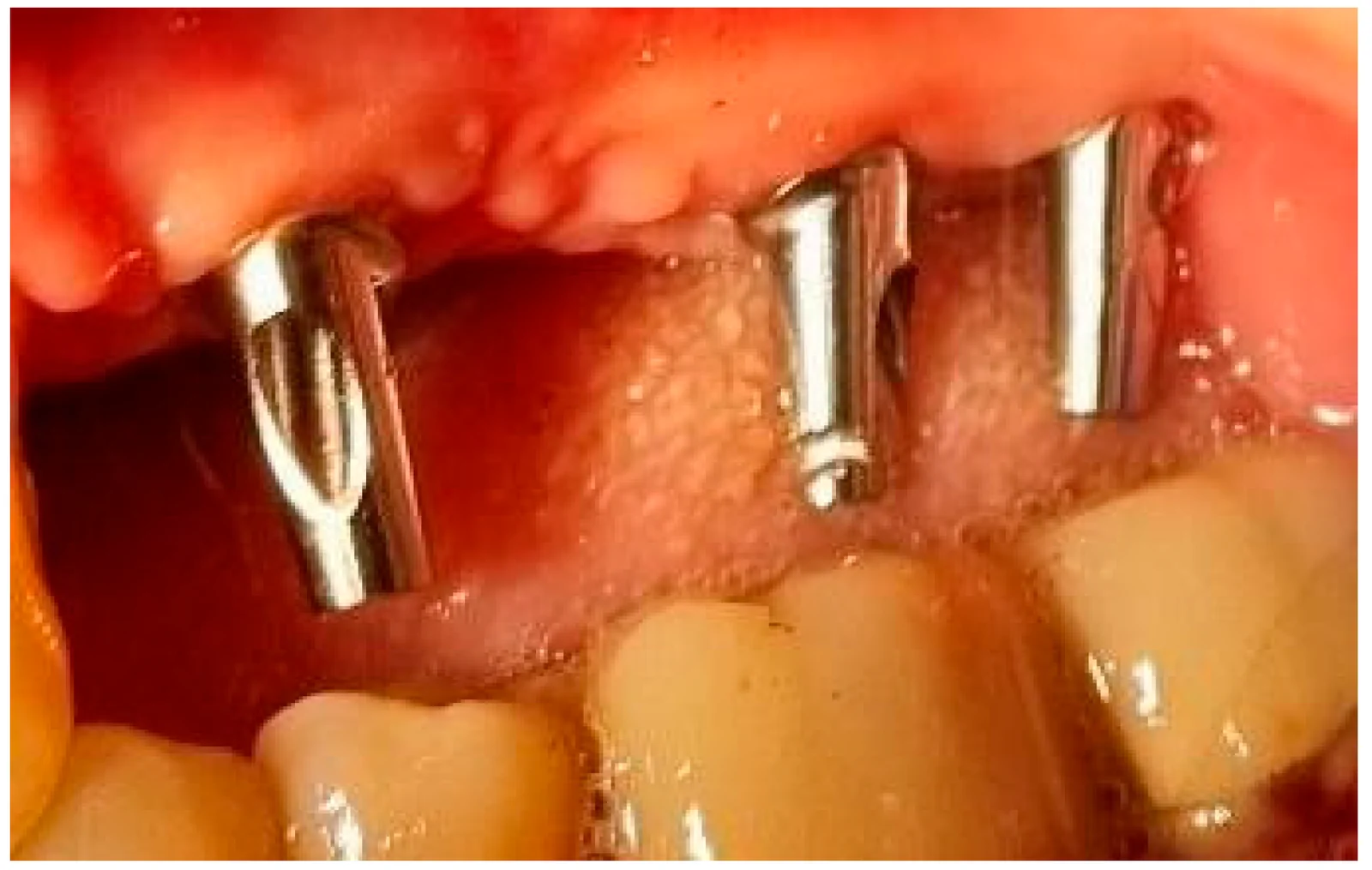
Placement of the standardized prosthetic abutments; the abutments must be fixed as parallel as possible.

The second appointment consisted of suture removal, unscrewing the healing abutments (Figure 6), placing the prosthetic abutments (Figure 7), scanning, and reapplying the healing abutments.

**Figure 8:**
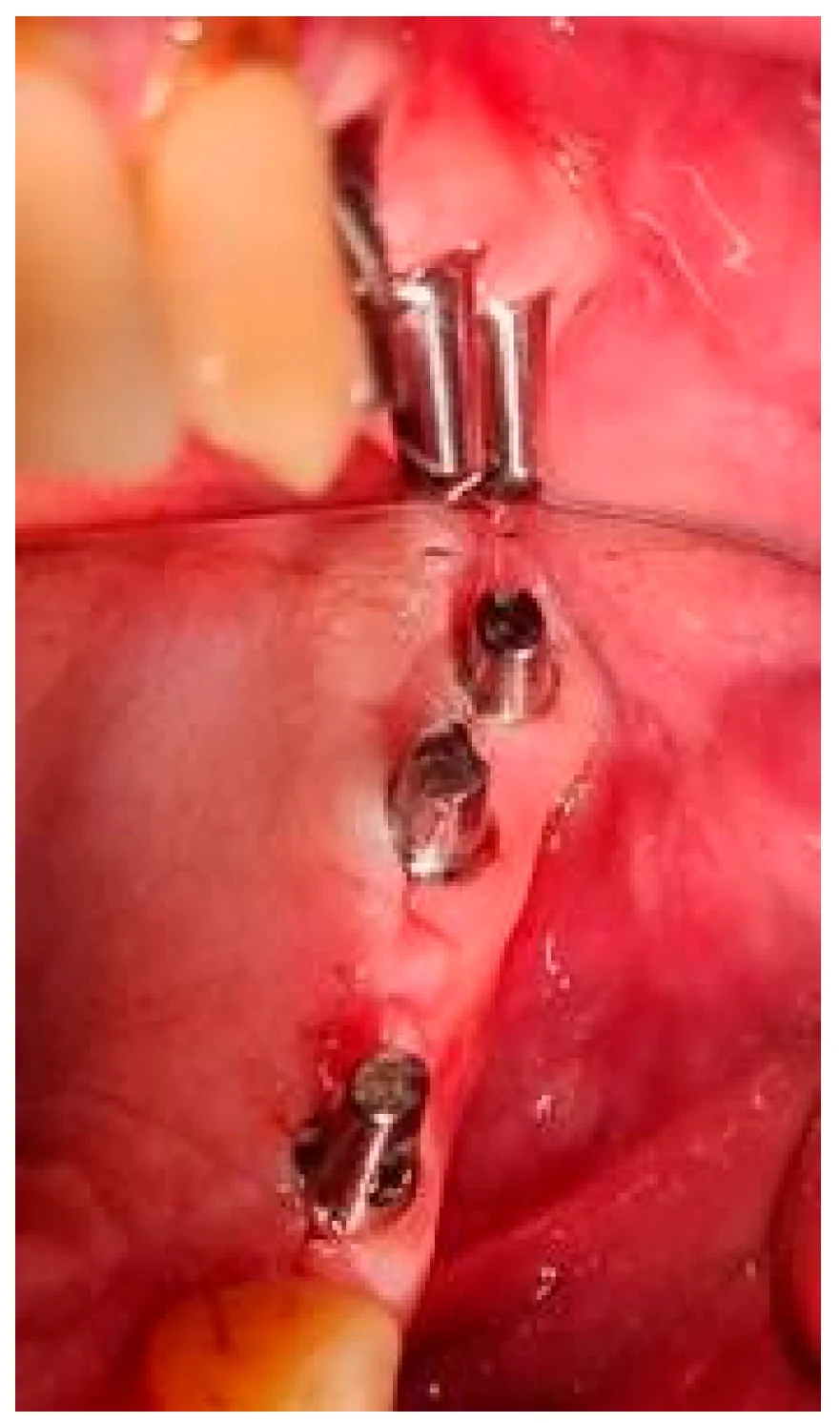
The shoulders are partially covered by the gingiva; a small gingivectomy is required.

**Figure 9:**
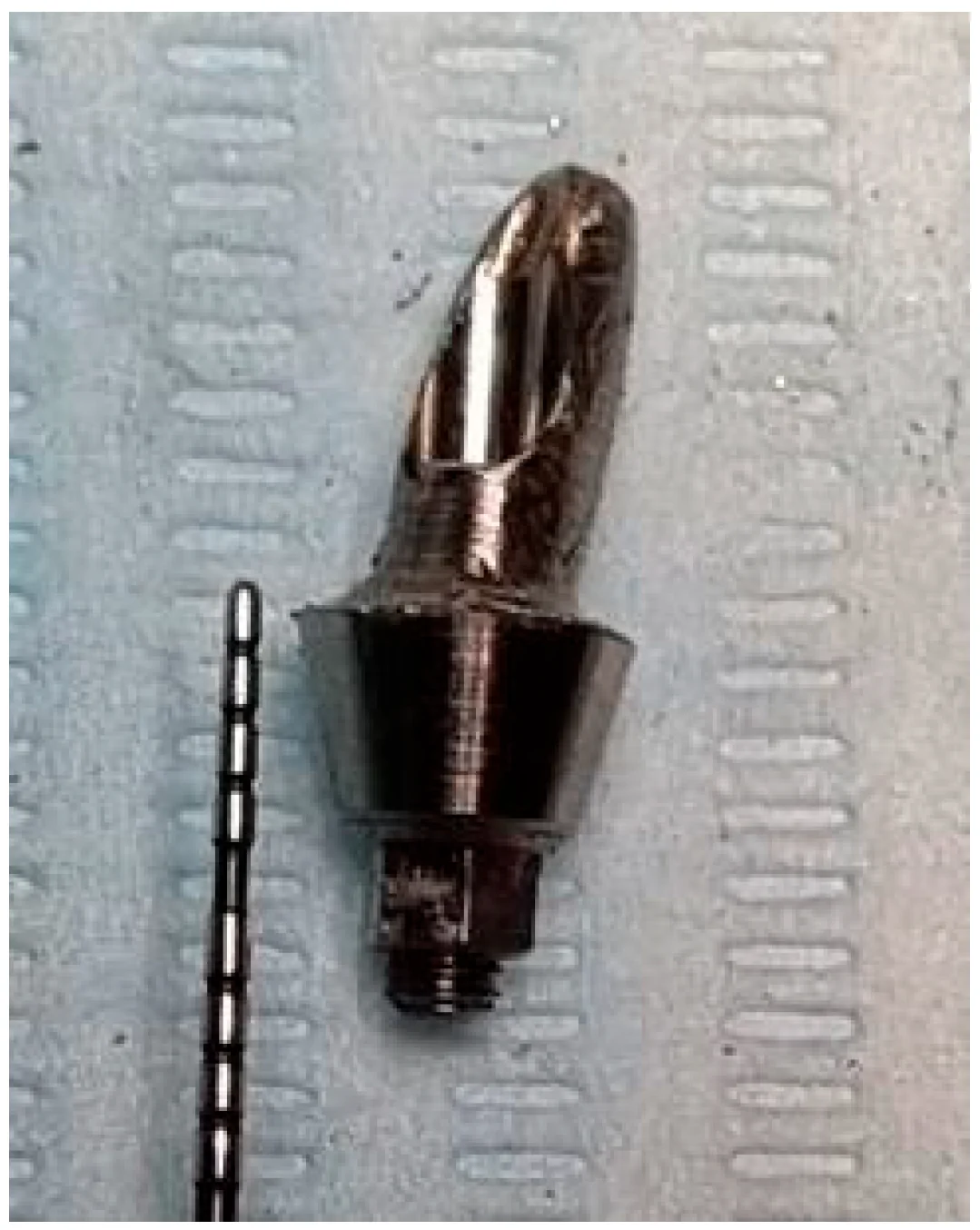
The 3 mm height of the prosthetic abutment shoulder.

**Figure 10:**
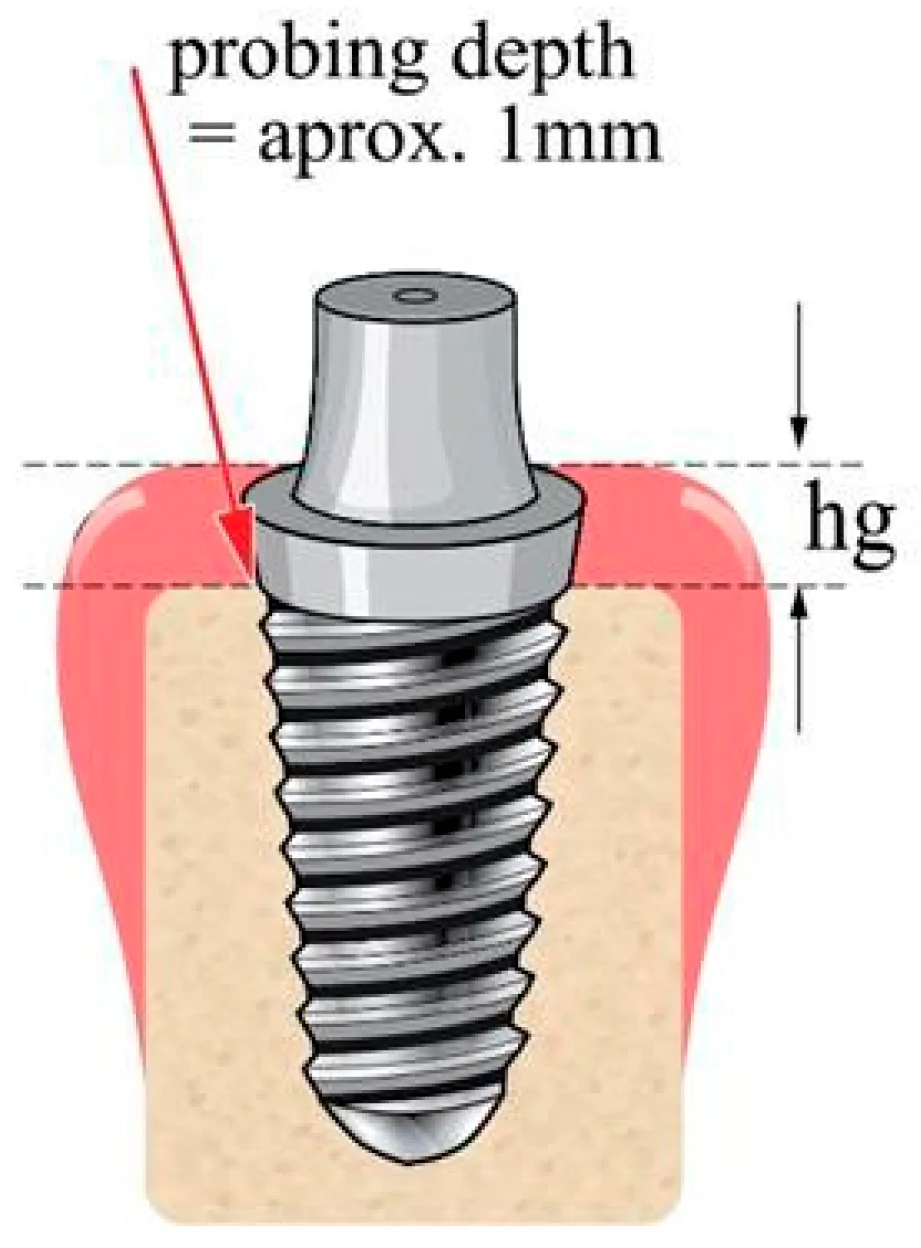
Bone-level implant: Preparation of the abutments in the posterior area coordinates the level of the shoulders (hg) and the gingival margin. Due to the tapered shape of the abutments used, which guides gingival contouring, the probing depth (red arrow) does not exceed 1 mm.

We used shouldered abutments, both straight and angled. The shoulder height ranged from 1 to 3 mm. Prosthetic abutments were selected according to the following criteria:

• In the insertion axis of the restoration, the abutments should be as parallel as possible and correspond to a normal occlusion (Figure 8).
• At this stage, the abutment shoulder remained visible to ensure reliable intraoral scanning (Figure 9). The maximum shoulder height was 3 mm (Figure 10). Later, before the final scan, this height could be modified, including by subgingival sinking in the esthetic zone.

After selecting the optimal prosthetic abutments, the intended reduction level was marked intraorally using a high-speed extra-hard cylindrical bur. Each abutment was then trimmed individually and extraorally mounted on laboratory analogs. Following trimming, the abutments were reinserted, and the faces requiring adjustment to achieve parallelism were identified and marked; these adjustments were subsequently performed individually extraorally.

**Figure 11:**
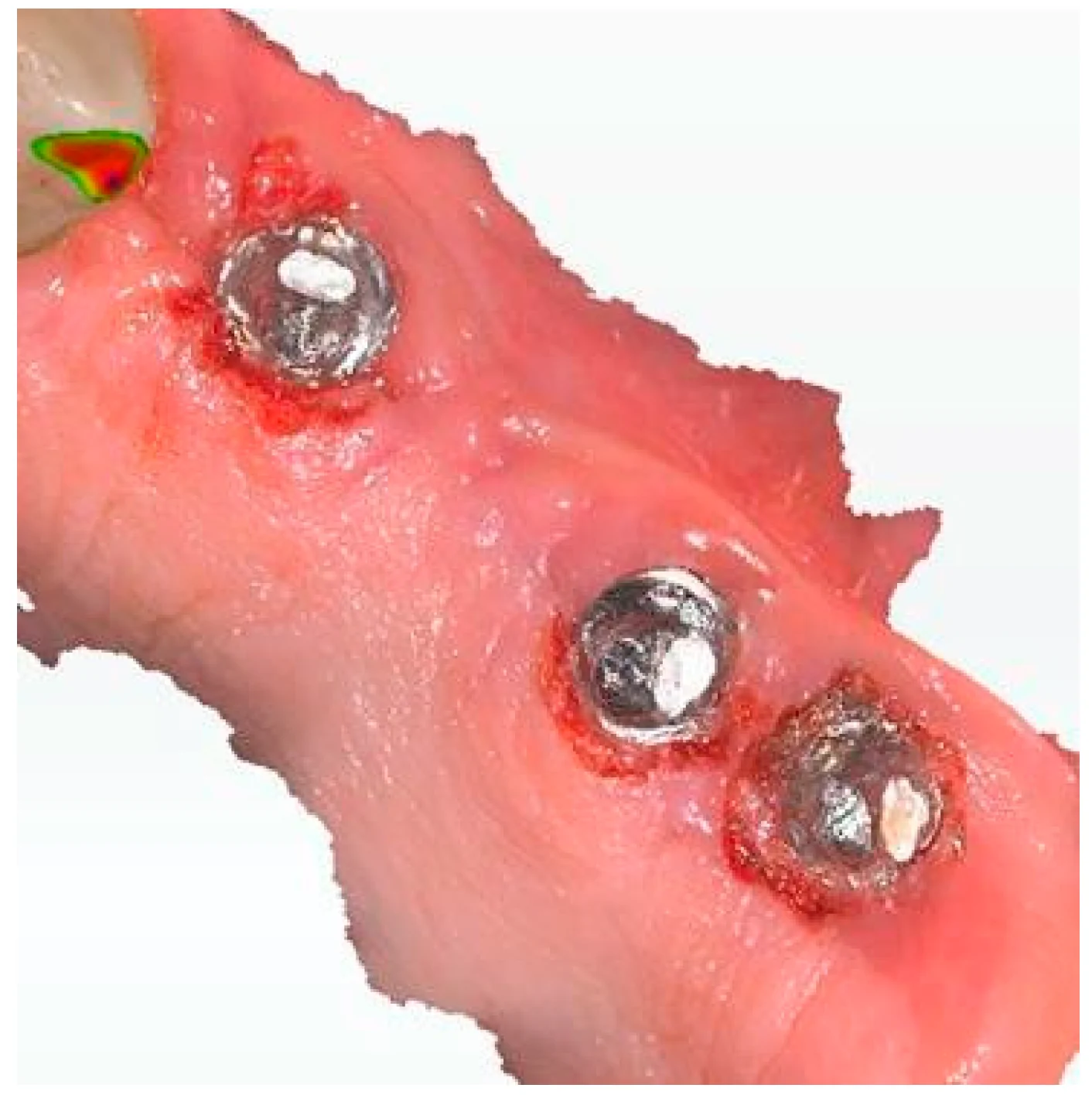
Intraoral scanning of the abutments to fabricate a PMMA (polymethyl methacrylate) provisional restoration; the gingiva surrounding the abutments shows evidence of recent contouring performed using a bur, electrocautery, or laser.

**Figure 12:**
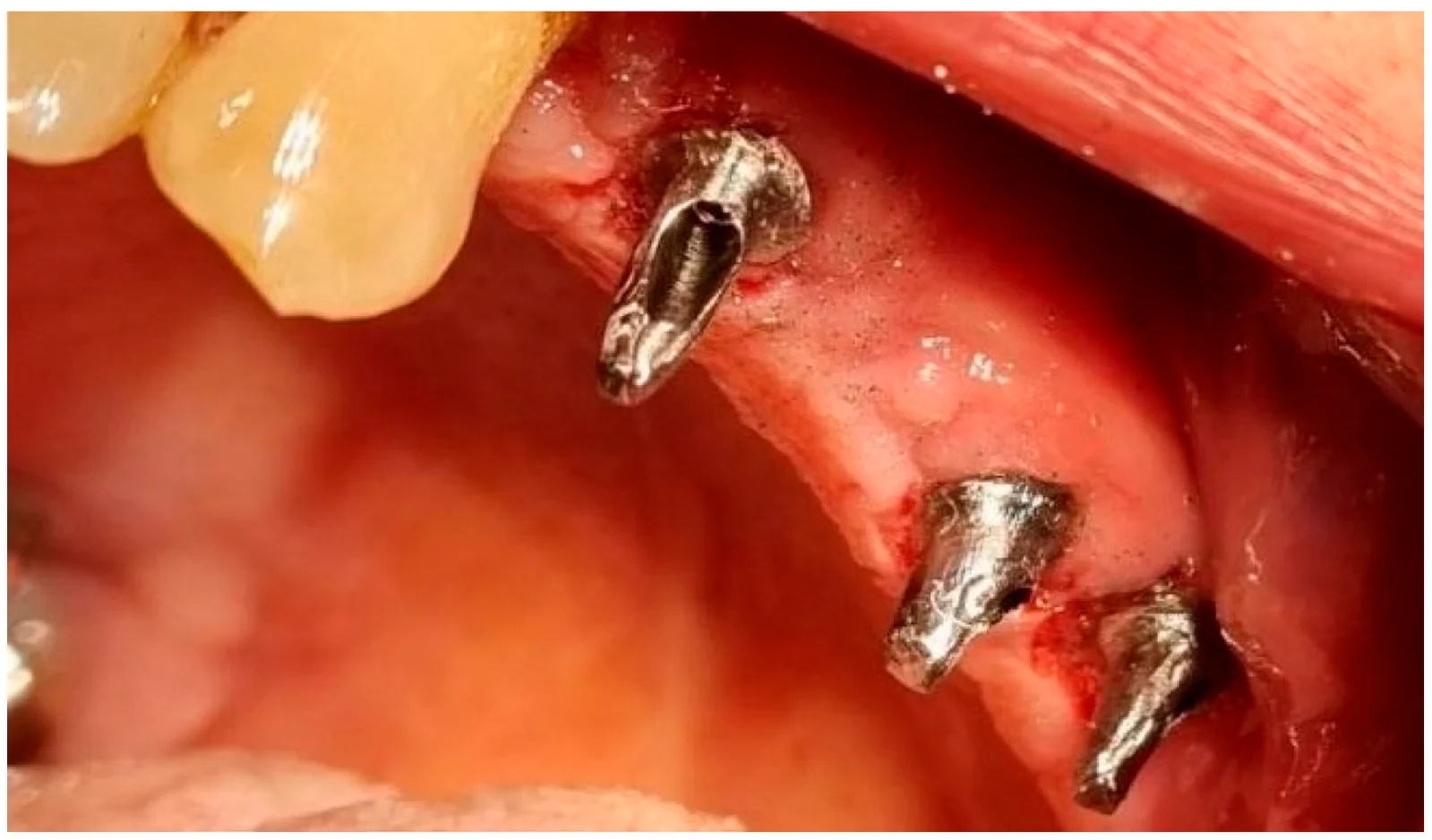
Intraoral photograph, vestibular view of abutments ready for the first scan.

At the end of the clinical session, with the prosthetic abutments seated intraorally, only minimal adjustment was performed using a diamond bur with large grain size under continuous irrigation. When the clinical appointment was near its scheduled end, abutment finishing was limited to an intermediate level (Figure 11 and Figure 12); definitive finishing would be completed during the next visit. An intraoral scan was made with the abutments in place. Because the abutments had been individually processed and presented distinct surface characteristics, individualized abutment geometry minimized the risk of scan recognition errors.

**Figure 13:**
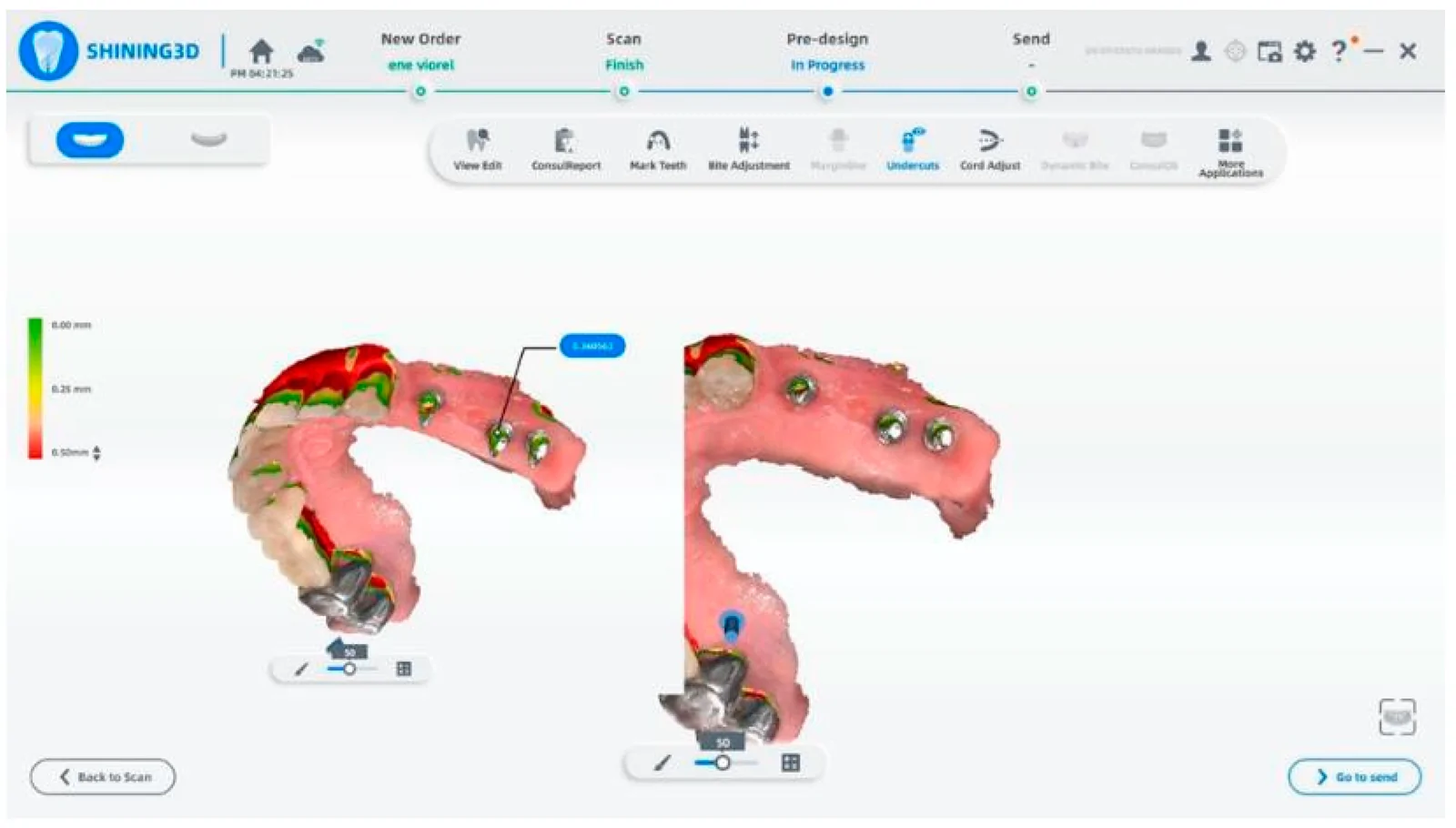
The “undercuts” command in the software allows visualization of undercut areas to a selected path of insertion; this path can also be modified to minimize potential undercuts.

The scanner software provides real-time verification of the prosthesis insertion axis when the clinician questions the preparation. Immediate feedback on abutment parallelism permits on-the-spot correction and immediate rescanning (Figure 13).

At the end of the appointment, abutments had a vestibular reference mark; then they were removed and stored in dedicated containers in the clinic.

**Figure 14:**
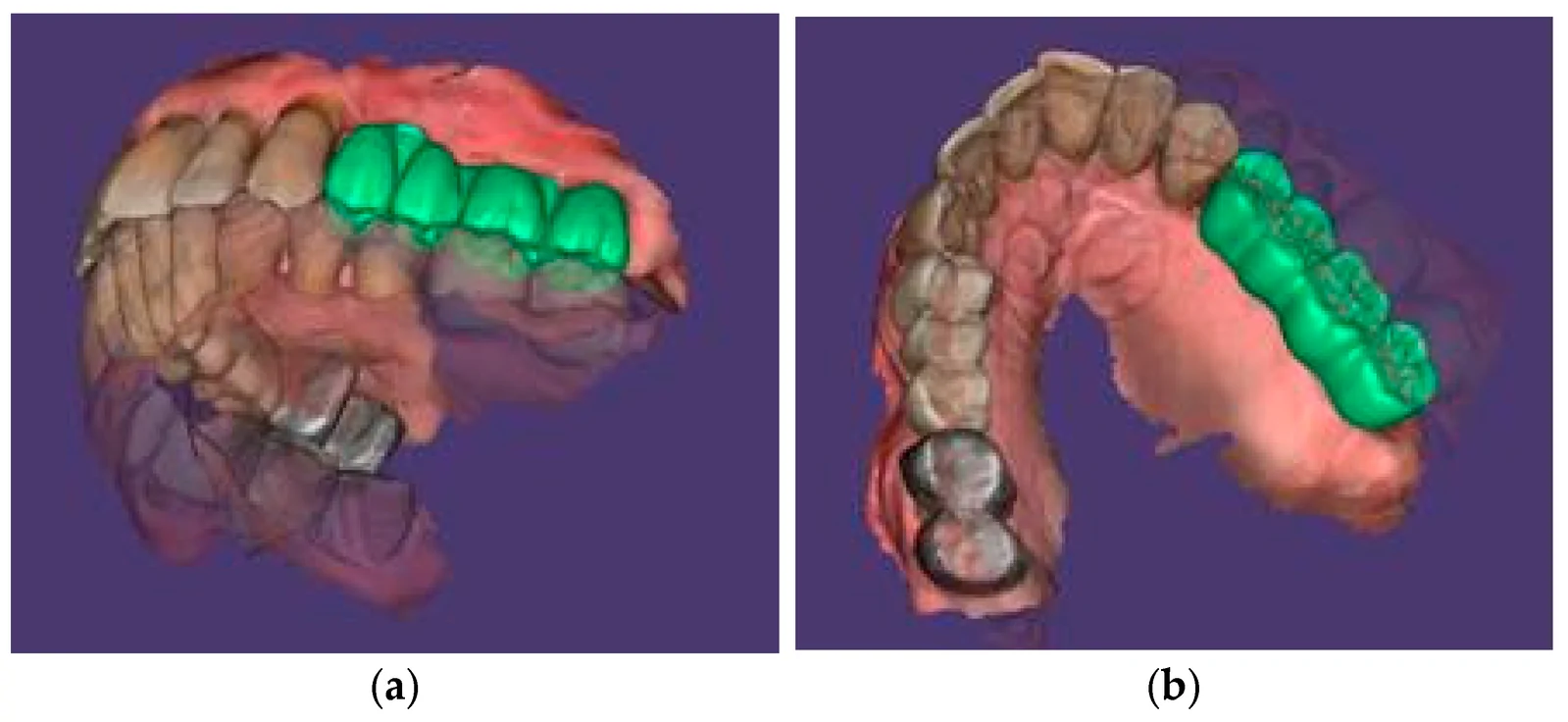
The Exocad project sent by the dental laboratory ((**a**) vestibular view and (**b**) occlusal view).

After completing the digital design, the technician sent the project to the clinician using exocad Dental CAD software3.2 Elefsina (exocad GmbH, Darmstadt, Germany) (Figure 14). The clinician reviewed tooth contours, morphology, and their relationship to the edentulous ridge, requesting modifications when necessary.

#### 2.4.3. Provisional Restoration Phase

In the third appointment, the provisional restoration was therefore delivered; the prosthetic abutments were placed, and then the prosthesis was inserted. Minor discrepancies in insertion, when present, were corrected at the abutment level, while the provisional restoration served as an additional verification of parallelism and passive fit [14].

Once insertion was achieved, the fit of the restorations on the shoulders, occlusion, convexities and contact points, relation to the gingiva and passivity were checked. The provisional restoration allowed hygienic access and functional evaluation. The provisional restoration should be designed to closely match the final restoration. Any modification made or required to the restoration was recorded in the chart and subsequently communicated to the technician.

**Figure 15:**
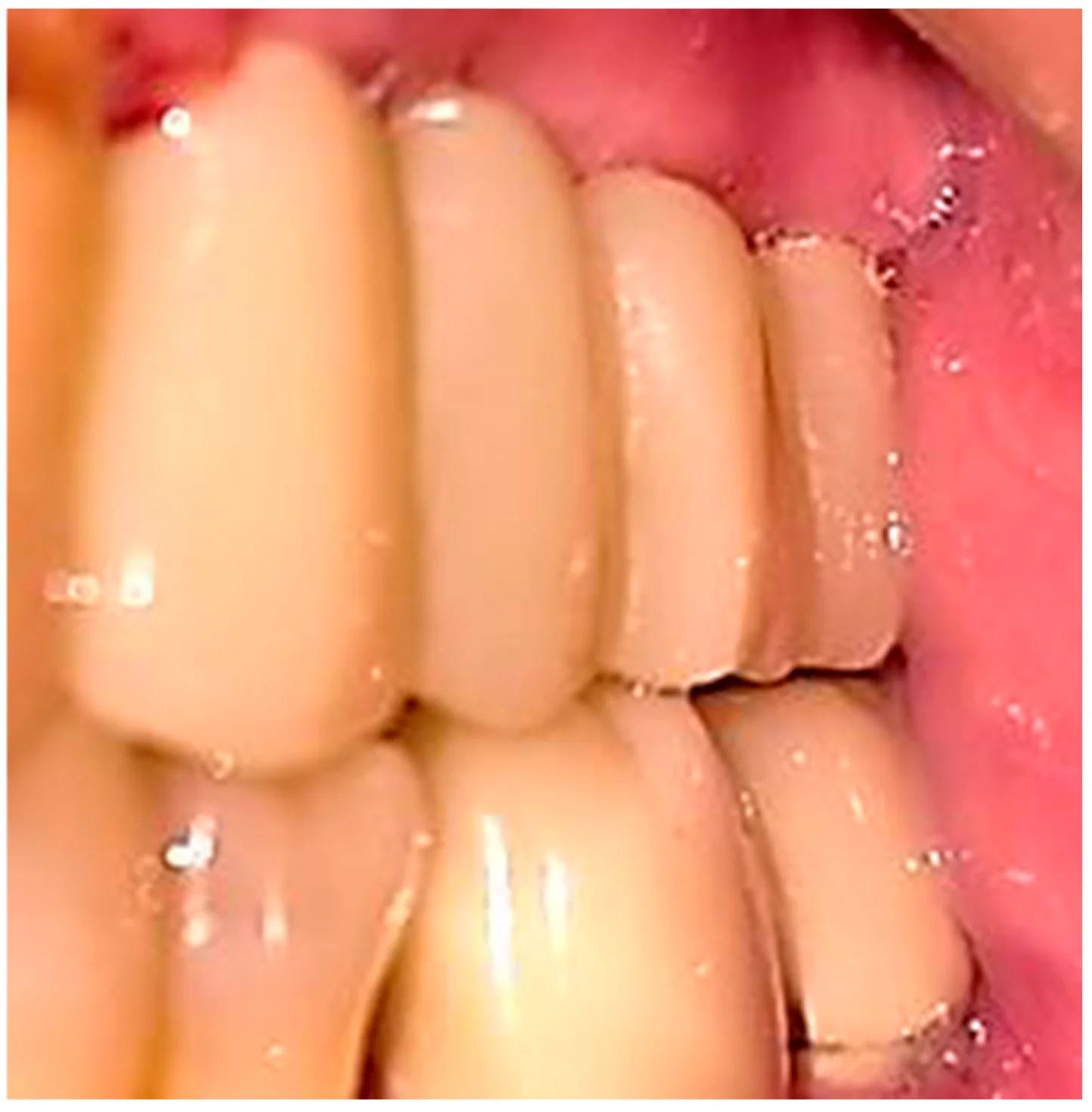
Intraoral photograph of the provisional bridge.

The provisional bridge was maintained in place for two weeks to allow final soft-tissue maturation and to perform a functional trial (Figure 15).

**Figure 16:**
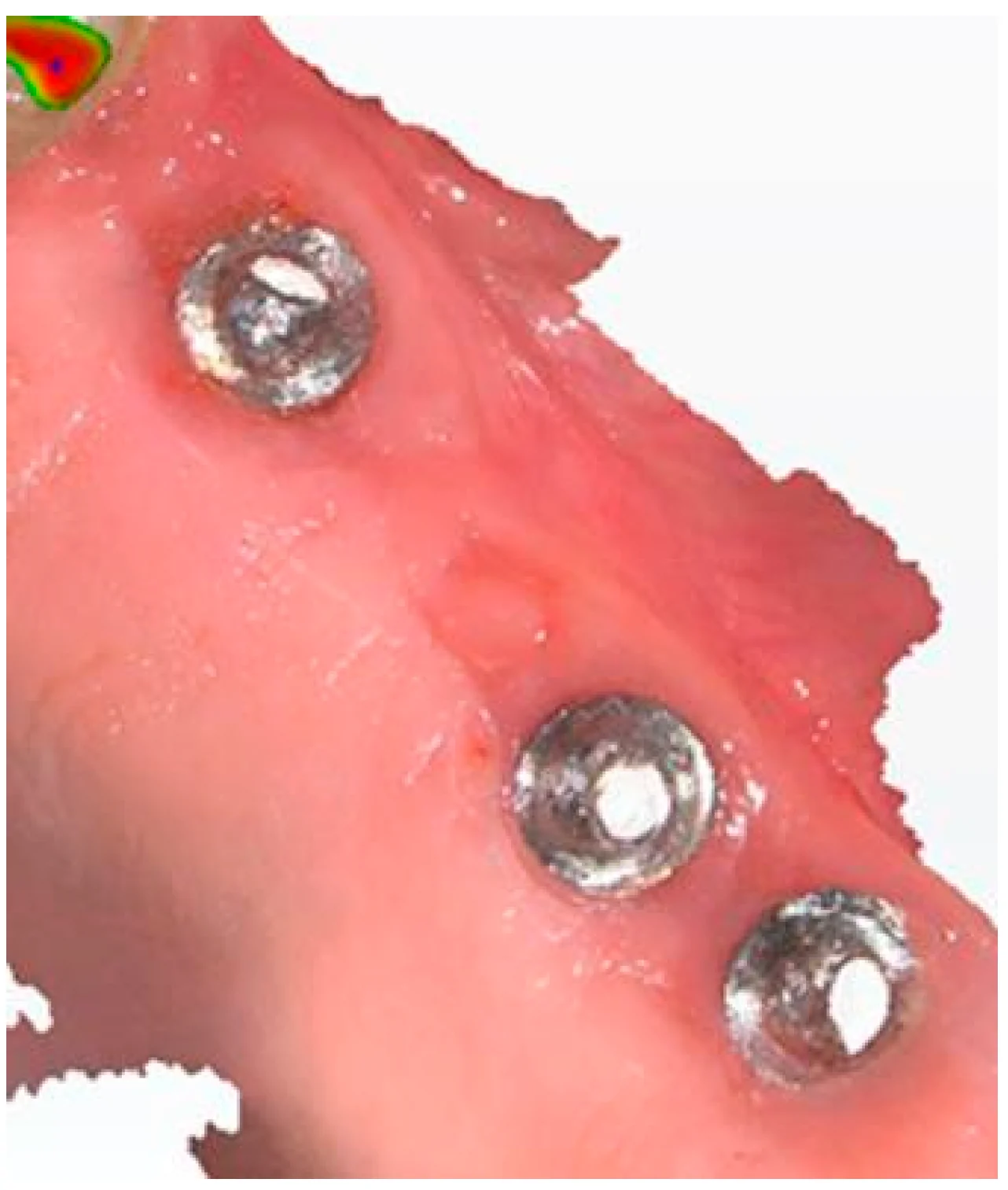
Intraoral scanning for fabrication of the definitive zirconia restoration. Complete healing and contouring of the marginal gingiva can be observed.

**Figure 17:**
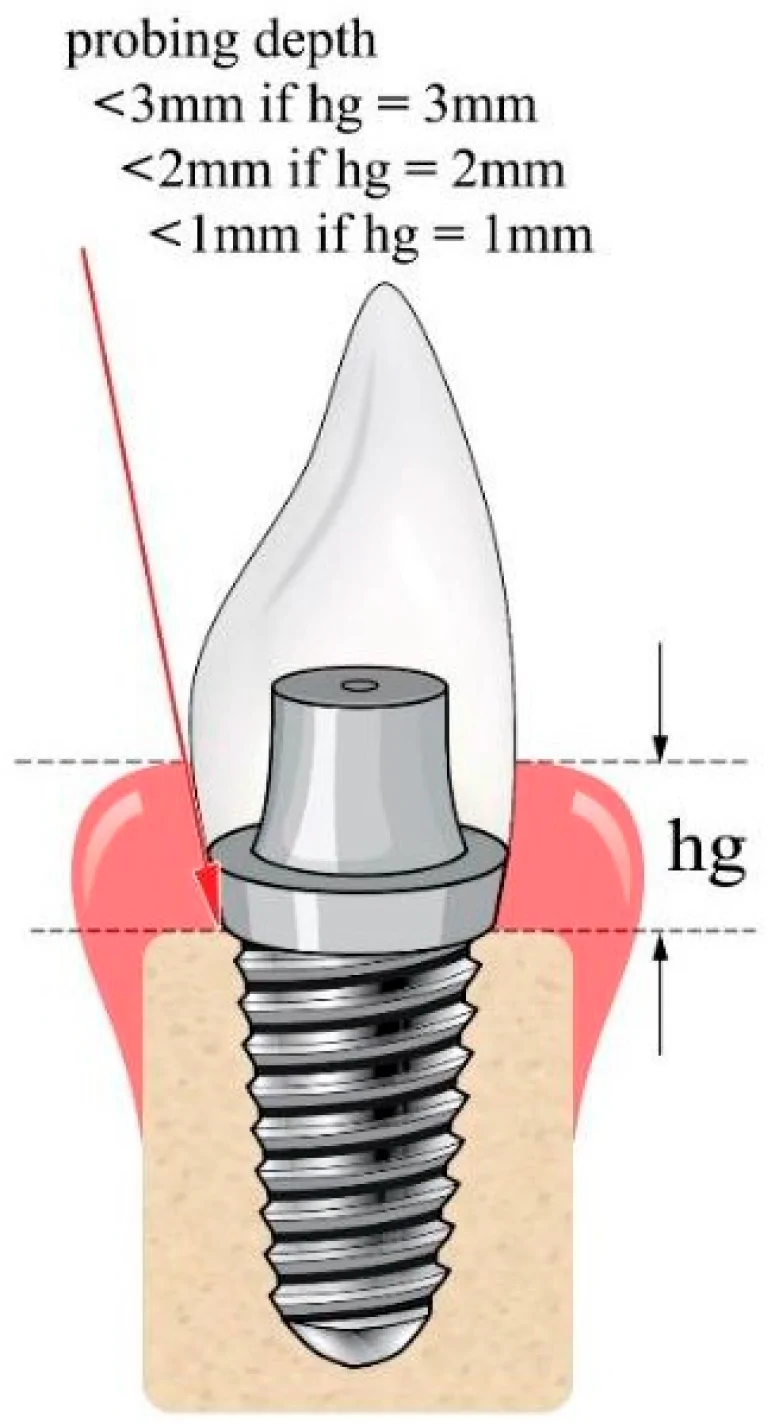
Bone-level implant in the anterior region; the abutment shoulder is positioned subgingivally before the final scan. After placement of the definitive crown, the probing depth depends on the gingival height, according to the rule illustrated in the figure under normal, healthy conditions.

At the fourth visit, the prosthetic abutments were definitively finished, with particular attention to shoulder height. The soft tissue had matured satisfactorily (Figure 16). Shoulders were positioned at the gingival margin in non-esthetic areas or were adjusted to lie 1–2 mm subgingivally in the esthetic zone (Figure 17); these adjustments were performed extraorally using the previously described burs. The restoration was inspected for pressure points, soft-tissue inflammation, and food-retentive areas. Patient feedback regarding comfort and esthetics was recorded.

#### 2.4.4. Definitive Restoration Delivery

Then the final scan was performed. If the provisional restoration had been modified, it was also scanned. All the data were sent to the laboratory. If no modifications were made, the final restorations were copies of the provisional restorations (already functionally tested).

**Figure 18:**
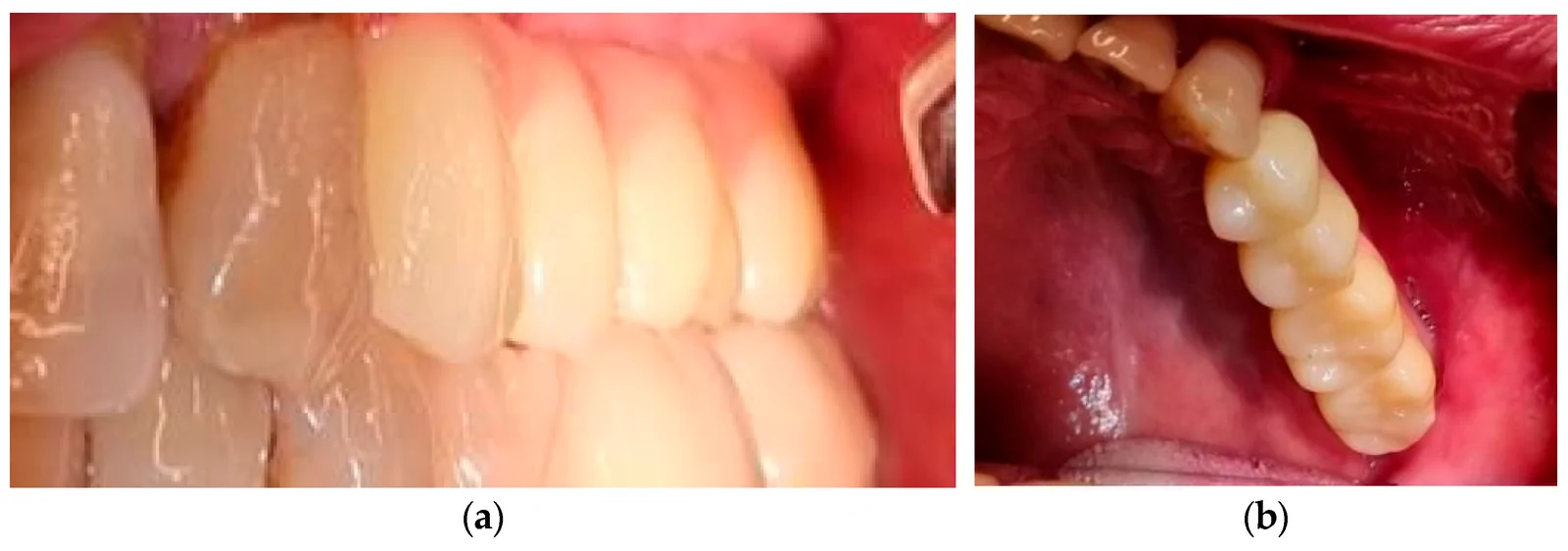
The definitive zirconia bridge after cementation ((**a**) vestibular view and (**b**) occlusal view).

**Figure 19:**
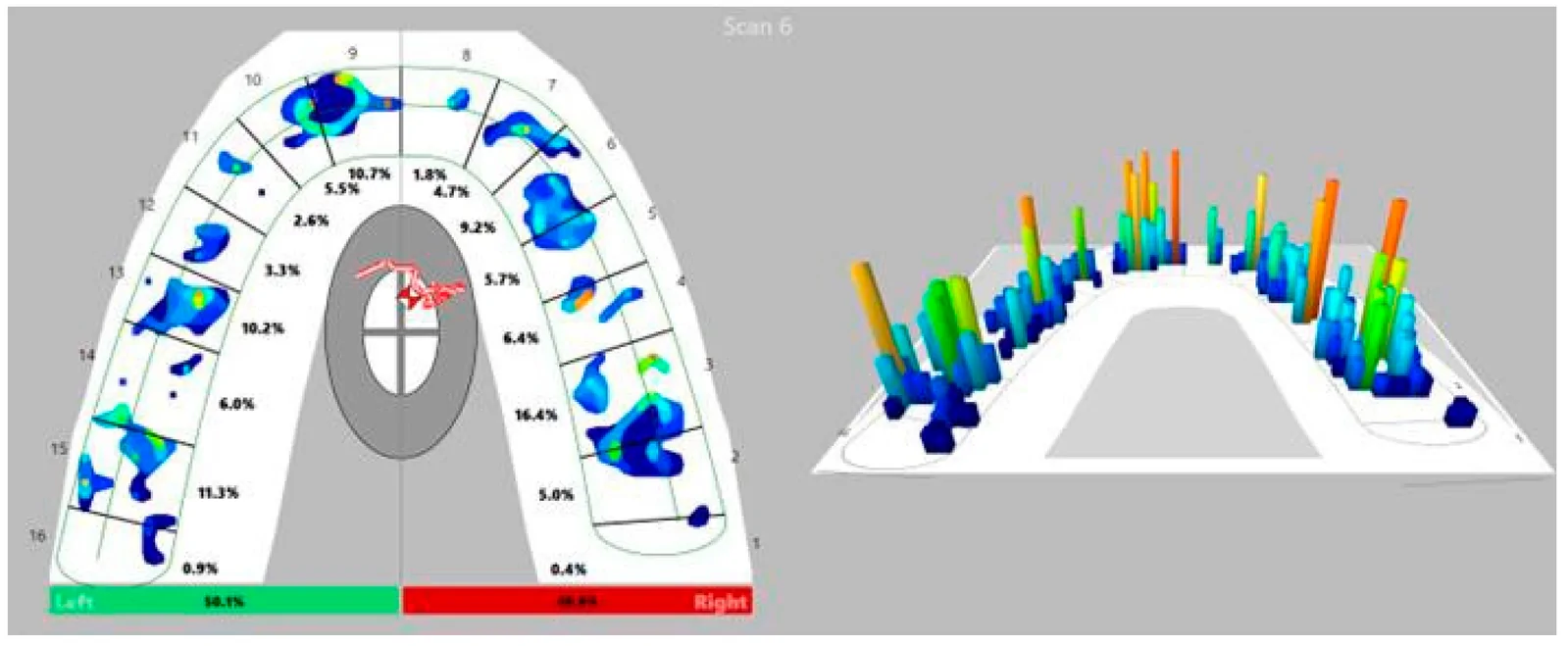
Occlusal adjustment performed using the T-Scan system.

The fifth and final appointment could be performed as early as 24 h after the final scan. The definitive restoration was an improved copy of the provisional. The definitive restoration reproduced the validated provisional design. All aspects were checked (contact points, convexities, fit on the shoulders, passivity, relation to the gingiva, color, occlusion), and the patient’s consent for definitive cementation was obtained. The screws were tightened with a torque wrench to 35 Ncm, the screw channels were protected with PTFE (Teflon) tape, and the restoration was prepared for cementation. Cementation was performed with dual-cure resin cement under a protocol aimed at minimizing residual cement (Figure 18), followed by final occlusal verification using the T-Scan Novus system (Tekscan Inc., Norwood, MA, USA) (Figure 19).

The sequence of clinical appointments and the corresponding stages of the progressive gingival digital workflow are summarized in the Graphical Abstract.

### 2.5. Statistical Analysis

Statistical analyses were performed using IBM SPSS Statistics 29 (IBM Corp., Armonk, NY, USA). Continuous variables were expressed as mean ± standard deviation or median and range, whereas categorical variables were reported as frequencies and percentages.

Because of the retrospective exploratory pilot design, no a priori sample size calculation was performed. Following peer review, exploratory post hoc power analyses were conducted using G*Power 3.1.9.7 (Heinrich Heine University Düsseldorf, Düsseldorf, Germany) (two-tailed tests, α = 0.05), indicating limited statistical power to detect the small intergroup differences observed [42].

Normality of data distribution was assessed using the Shapiro–Wilk and Kolmogorov–Smirnov tests together with graphical inspection. As several variables did not meet parametric assumptions, non-parametric methods were applied where appropriate.

Associations between continuous variables were evaluated using Spearman’s correlation coefficient. Intergroup comparisons were performed using the Mann–Whitney U test, while categorical associations were analyzed using the Chi-square test. Effect sizes were estimated using rank-biserial correlation coefficients (r), Phi (ϕ), or Cramer’s V coefficients (V). Binary logistic regression models were used to explore factors associated with bleeding on probing and marginal bone resorption. Model fit was evaluated using the Hosmer–Lemeshow test and pseudo-R^2^ coefficients (Cox and Snell; Nagelkerke). Receiver operating characteristic (ROC) analysis was used to assess model discrimination. Because several patients received more than one implant, supplementary analyses were performed using generalized estimating equations (GEE) with a binomial distribution and logit link function, applying an exchangeable working correlation structure and robust variance estimators while considering the patient as the clustering unit. These analyses were conducted to account for the potential lack of independence among implants placed in the same patient.

Linear regression analyses were additionally performed for selected outcomes after verification of assumptions of linearity, homoscedasticity, residual normality, and independence of errors (Durbin–Watson test).

Statistical significance was set at *p* < 0.05. Given the exploratory pilot design, analyses were performed at the implant level; potential within-patient clustering was acknowledged as a methodological limitation.

Multicollinearity was assessed using variance inflation factors (VIF). Linearity of continuous predictors in the logit was evaluated using the Box–Tidwell procedure.

## 3. Results

The analysis included 161 implants placed in 39 patients (19 men, 20 women), of which 87 were bone-level implants (experimental group; 54.04%), and 74 were tissue-level implants (control group; 45.96%). Age, gingival phenotype, implant position, sex distribution, and implant duration were comparable between groups. Baseline demographic and clinical characteristics are summarized in Table 2.

The groups did not differ significantly with respect to the evaluated baseline characteristics, including age, sex distribution, gingival phenotype, implant position, and implant duration. Patient satisfaction was uniformly high in both groups. Most patients assigned the maximum VAS score of 10 for all evaluated domains, whereas two patients in each group reported a score of 9 due to minor esthetic concerns.

Biological and prosthetic complications recorded during follow-up are summarized in Table 3. Overall, the incidence of complications was low in both groups. One fracture of a provisional restoration was observed in the control group. In the experimental group, one case each of decementation, loss of contact point, transient phonation adaptation problems, and prosthesis-related pain was recorded. No implant failures or screw-loosening events occurred in either group during the observation period.

**Figure 20:**
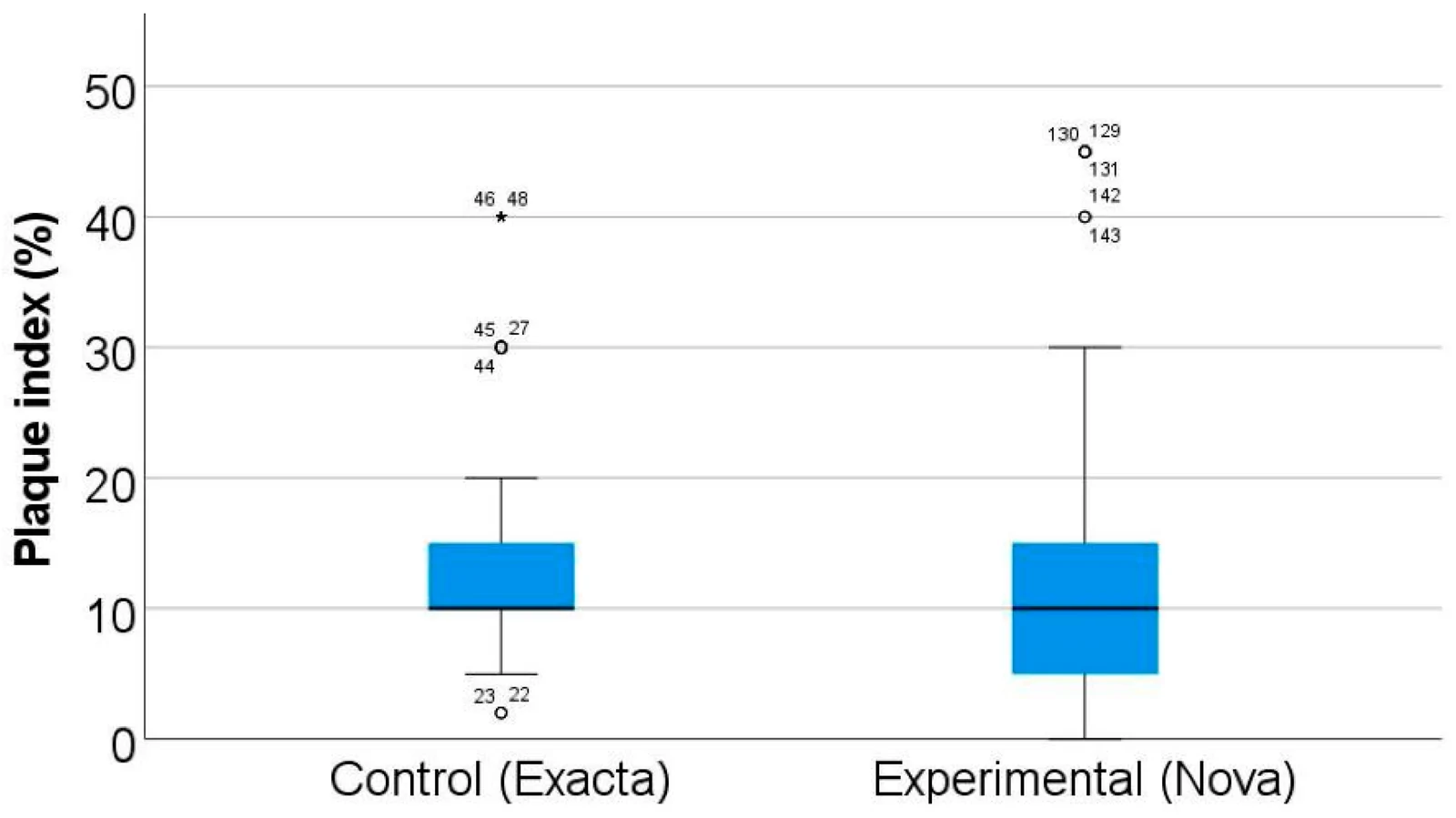
Distribution of the Plaque index in the two groups. Outliers are shown as individual dots. * values are expressed as percentages of implants included in each study group.

Diagnostic analyses revealed no evidence of problematic multicollinearity among the included predictors (VIF range: 1.06–1.27). In addition, the Box–Tidwell procedure did not identify significant departures from the linearity assumption for continuous predictors. The plaque index ranged between 2% and 40%, with a mean value of 13.57 ± 8.14% and a median of 10% (experimental group). In the control group, the plaque index ranged between 0% and 45%, with a mean value of 13.32 ± 10.11% and a median of 10%. No statistically significant differences were found between the two groups (*p* = 0.304, r = −0.081) (Figure 20).

**Figure 21:**
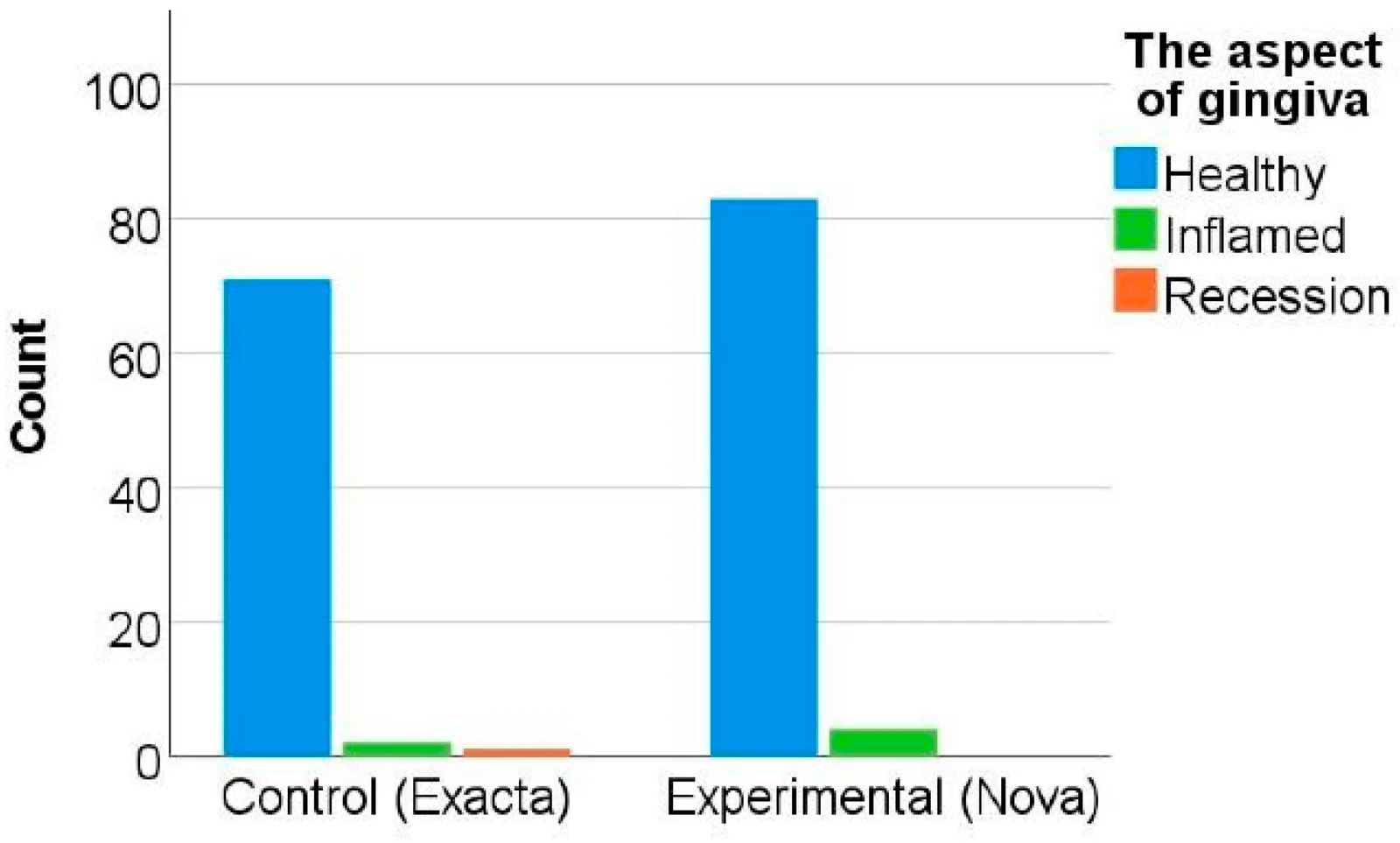
Gingival condition in the two groups. Values represent the number of implants in each category.

Gingival condition was predominantly normal in both groups, with no statistically significant differences observed between them (*p* = 0.458, V = 0.090). In the experimental group, 95.40% were normal (*n* = 83), while 4.60% showed inflammation (*n* = 4). In the control group, 95.95% were normal (*n* = 71), 2.70% were inflamed (*n* = 2), and 1.35% presented with recession (*n* = 1) (Figure 21).

**Figure 22:**
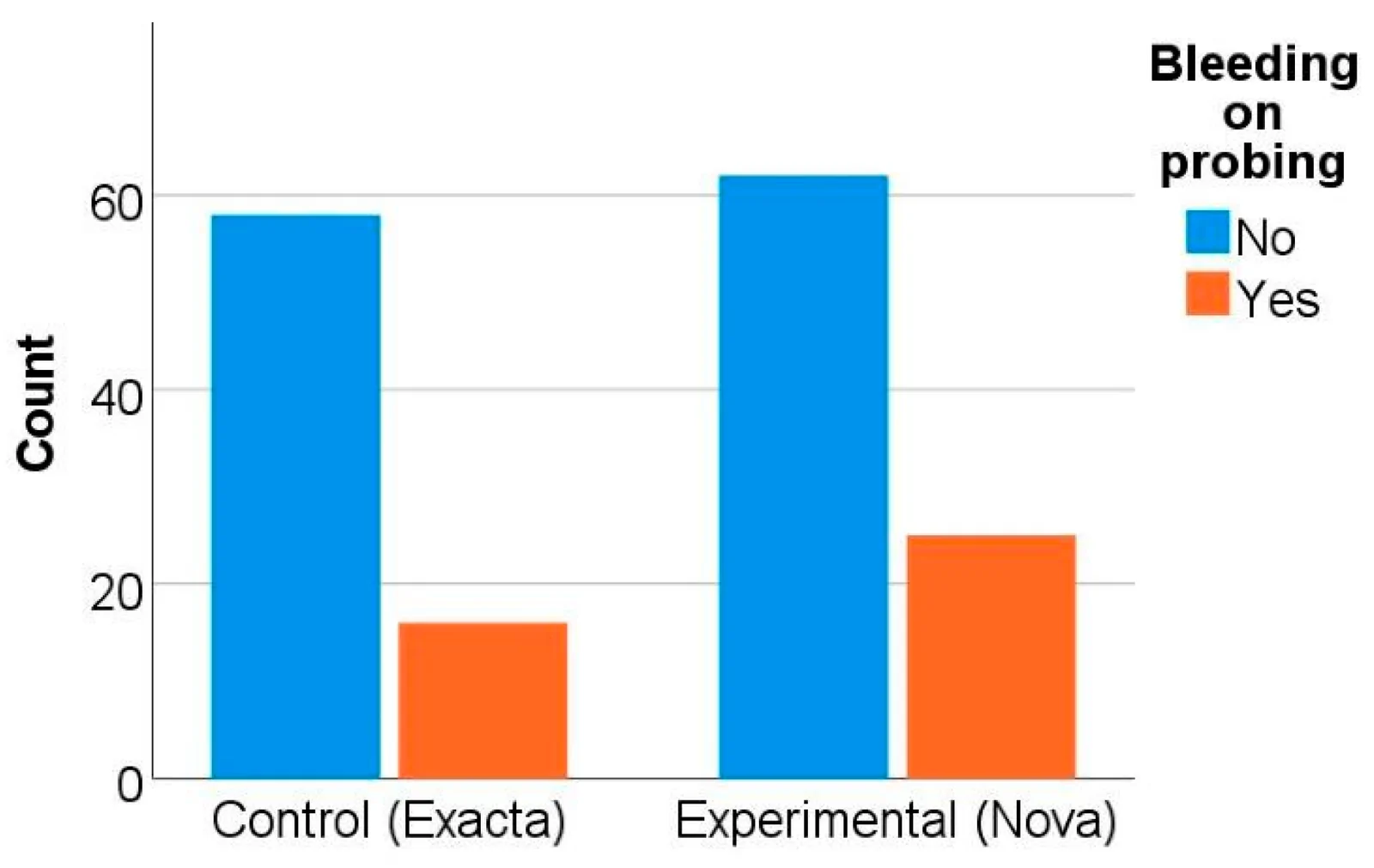
Distribution of bleeding on probing between the experimental and control groups. Bleeding on probing was observed in 28.74% of implants (*n* = 25) in the experimental group and in 21.62% of implants (*n* = 16) in the control group. Values represent the number of implants in each category.

Bleeding on probing occurred on average in 1 out of 4 implants, slightly more frequently in the experimental group compared to the control group (28.74% vs. 21.62%), without constituting a statistically significant difference between groups (*p* = 0.302, ϕ = 0.080) (Figure 22).

Vestibular sulcus

In the experimental group, the depth of the vestibular sulcus ranged between 1 and 7 mm, with a mean value of 2.26 ± 1.32 mm and a median of 2 mm.

In the control group, the depth of the vestibular sulcus ranged between 0 and 7 mm, with a mean value of 1.99 ± 1.27 mm and a median of 2 mm.

The depth of the vestibular sulcus did not differ significantly between the two groups (*p* = 0.310, r = 0.080) (Figure 22).

Oral sulcus

In the experimental group, the depth of the oral sulcus ranged between 0 and 8 mm, with a mean value of 2.28 ± 1.42 mm and a median of 2 mm.

In the control group, the depth of the oral sulcus ranged between 0 and 7 mm, with a mean value of 1.85 ± 1.18 mm and a median of 2 mm.

**Figure 23:**
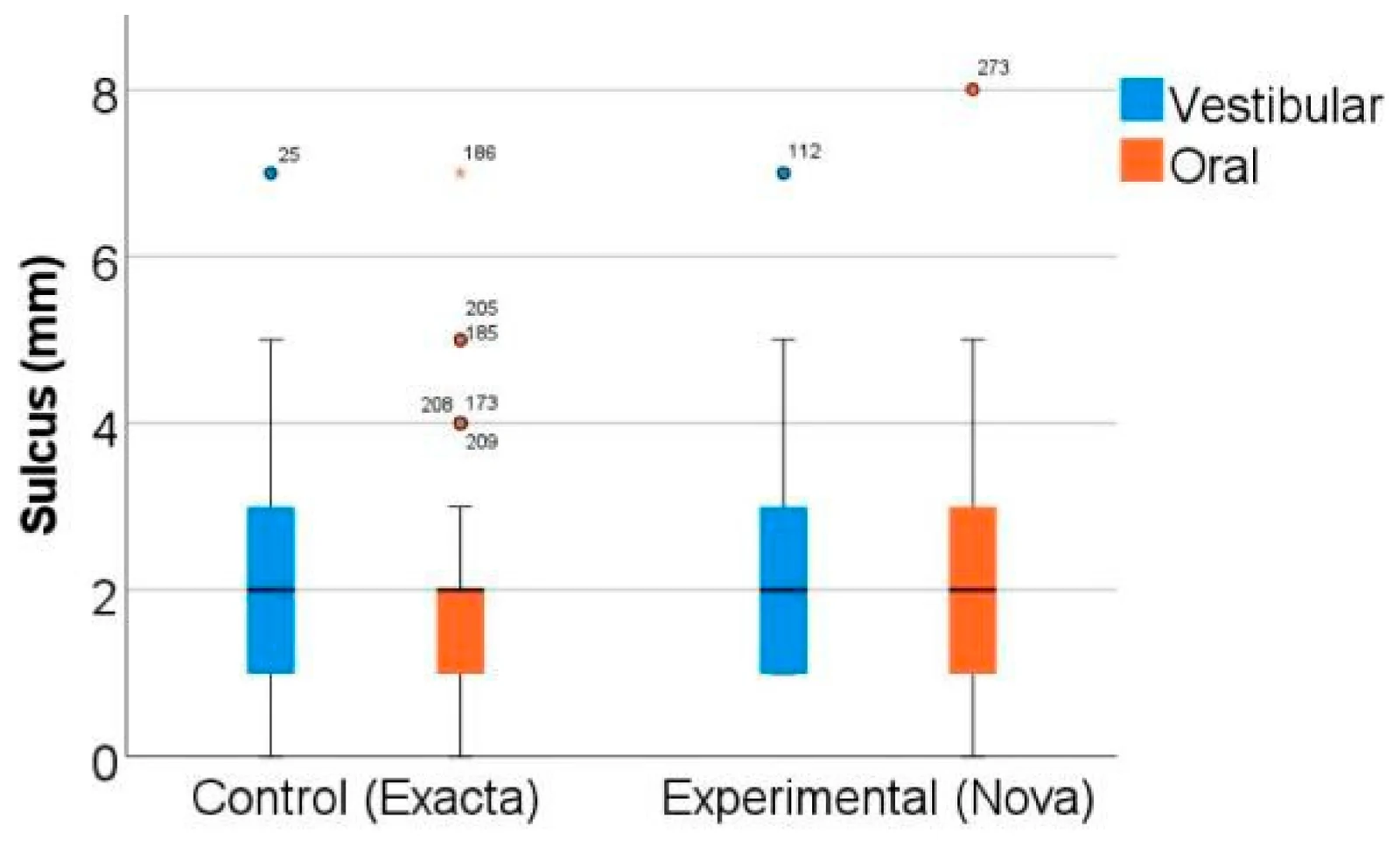
Distribution of peri-implant sulcus depth in the two groups (vestibular and oral measurements).

The depth of the oral sulcus did not differ significantly between the two groups (*p* = 0.064, r = 0.146) (Figure 23).

Bone resorption

Bone resorption was observed in approximately 11–12% of implants. In the experimental group, resorption occurred in 11.49% of cases (*n* = 10). The extent of resorption ranged between 0.50 and 5.50 mm, with a mean value of 1.65 ± 1.42 mm and a median of 1.25 mm.

In the control group, resorption occurred in 12.16% of cases (*n* = 9). The extent of resorption ranged between 1 and 3 mm, with a mean value of 1.39 ± 0.70 mm and a median of 1 mm.

**Figure 24:**
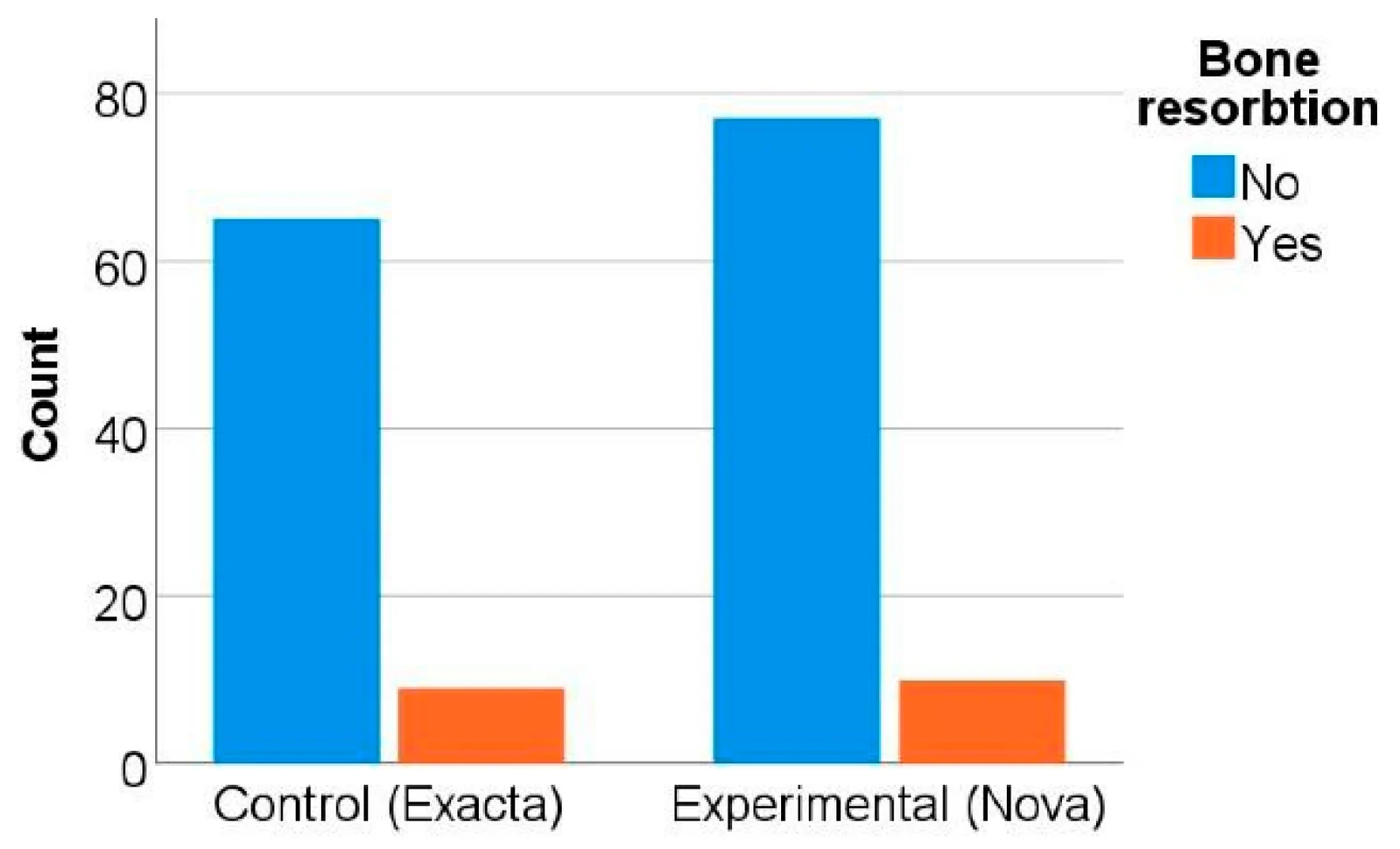
Radiographic assessment of marginal peri-implant bone loss in the two groups. Values represent the number of implants in each category.

From a statistical perspective, no significant differences were found between the groups, either in terms of resorption frequency (*p* = 0.896, ϕ = 0.010) or magnitude (in mm) (*p* = 0.842, r = 0.061) (Figure 24).

To account for the potential clustering of implants within the same patient, supplementary generalized estimating equations (GEE) analyses were performed. The results remained consistent with those obtained using logistic regression. Plaque index (OR = 1.075; *p* < 0.001), vestibular probing depth (OR = 1.508; *p* = 0.029), and implant age (OR = 1.083; *p* < 0.001) remained significantly associated with bleeding, whereas implant type was not significantly associated with bleeding (OR = 0.615; *p* = 0.298).

Correlation between bleeding and plaque index

In both groups, bleeding was associated with a higher plaque index.

Experimental Group: Cases with bleeding showed a significantly higher plaque index (20.22 ± 12.22%) compared to those without bleeding (10.54 ± 7.61%) (U = 1210, Z = 4.135, *p* < 0.001).

Control Group: Cases with bleeding also showed a significantly higher plaque index (18.00 ± 11.27%) compared to those without bleeding (12.34 ± 6.66%) (U = 613.500, Z = 2.092, *p* = 0.036).

Correlation between bleeding and vestibular sulcus

In the experimental group, cases with bleeding had a significantly deeper vestibular sulcus (3.12 ± 1.56 mm) compared to those without bleeding (1.91 ± 1.03 mm) (U = 1130, Z = 3.441, *p* < 0.001).

In the control group, cases with bleeding had a deeper vestibular sulcus (2.59 ± 1.69 mm) compared to those without bleeding (1.83 ± 1.09 mm), though the difference was not statistically significant (*p* = 0.169).

Correlation between bleeding and oral sulcus

Experimental Group: Cases with bleeding had a significantly deeper oral sulcus (3.08 ± 1.63 mm) compared to those without bleeding (1.95 ± 1.20 mm) (U = 1119.50, Z = 3.319, *p* < 0.001).

Control Group: Cases with bleeding had a significantly deeper oral sulcus (2.56 ± 1.71 mm) compared to those without bleeding (1.66 ± 0.90 mm) (U = 605.50, Z = 1.969, *p* = 0.049).

Binary logistic regression model for the occurrence of bone resorption

A binary logistic regression analysis was performed to evaluate factors associated with the occurrence of bone resorption, including implant type, plaque index, and gingival biotype, with the model adjusted for age and sex. The model was not statistically significant (*p* = 0.760), indicating that the included variables did not contribute significantly to explaining the occurrence of bone resorption. The explanatory capacity of the model was low, and its performance in case classification was limited. In the multivariate analysis, none of the included variables were significantly associated with bone resorption (*p* > 0.05 for all variables). Thus, neither implant type (OR = 0.874, 95% CI: 0.330–2.314, *p* = 0.874) nor the other specified parameters were associated with the occurrence of bone resorption, suggesting that other factors beyond those captured in the present exploratory analysis most likely contribute to its development. The results of the multivariable analysis should be interpreted cautiously because of the limited number of bone resorption cases observed.

Binary logistic regression model for the occurrence of bleeding

An exploratory multivariable logistic regression model was constructed to identify independent predictors of bleeding occurrence. The model included implant type, plaque index, vestibular sulcus dimension, bone resorption, and implant duration, adjusted for age and sex. The model was statistically significant (χ^2^ = 49.714, *p* < 0.001) and demonstrated moderate explanatory capacity (Cox & Snell R^2^ = 0.266, Nagelkerke R^2^ = 0.392). The Hosmer–Lemeshow goodness-of-fit test indicated good model fit to the data (*p* = 0.572). Overall accuracy was 80.7%, with high specificity (93.3%) and moderate sensitivity (43.9%).

In the multivariable model, bleeding on probing was significantly associated with:

• Plaque index (OR = 1.084, 95% CI: 1.032–1.139, *p* = 0.001): each 1% increase in plaque index was associated with an 8.4% increase in the likelihood of bleeding.
• Vestibular sulcus dimension (OR = 1.888, 95% CI: 1.295–2.753, *p* < 0.001): each 1 mm increase in vestibular sulcus depth was associated with an 88.8% increase in the likelihood of bleeding.
• Implant duration (OR = 1.074, 95% CI: 1.008–1.145, *p* = 0.026): each additional month of implant duration was associated with increased odds of bleeding.
• No statistically significant differences in bleeding on probing were detected between the two implant types (*p* = 0.545). Likewise, the presence of bone 518 resorption was not associated with bleeding on probing (*p* = 0.522).

To account for the inclusion of multiple implants in some patients, a generalized estimating equation (GEE) analysis was performed. The model confirmed the primary findings: bleeding on probing remained significantly associated with plaque index (OR = 1.075, 95% CI: 1.031–1.121, *p* < 0.001), vestibular sulcus dimension (OR = 1.508, 95% CI: 1.044–2.178, *p* = 0.029), and implant duration (OR = 1.083, 95% CI: 1.038–1.129, *p* < 0.001). Implant type was not significantly associated with bleeding on probing (*p* = 0.298), consistent with the results of the primary analysis.

**Figure 25:**
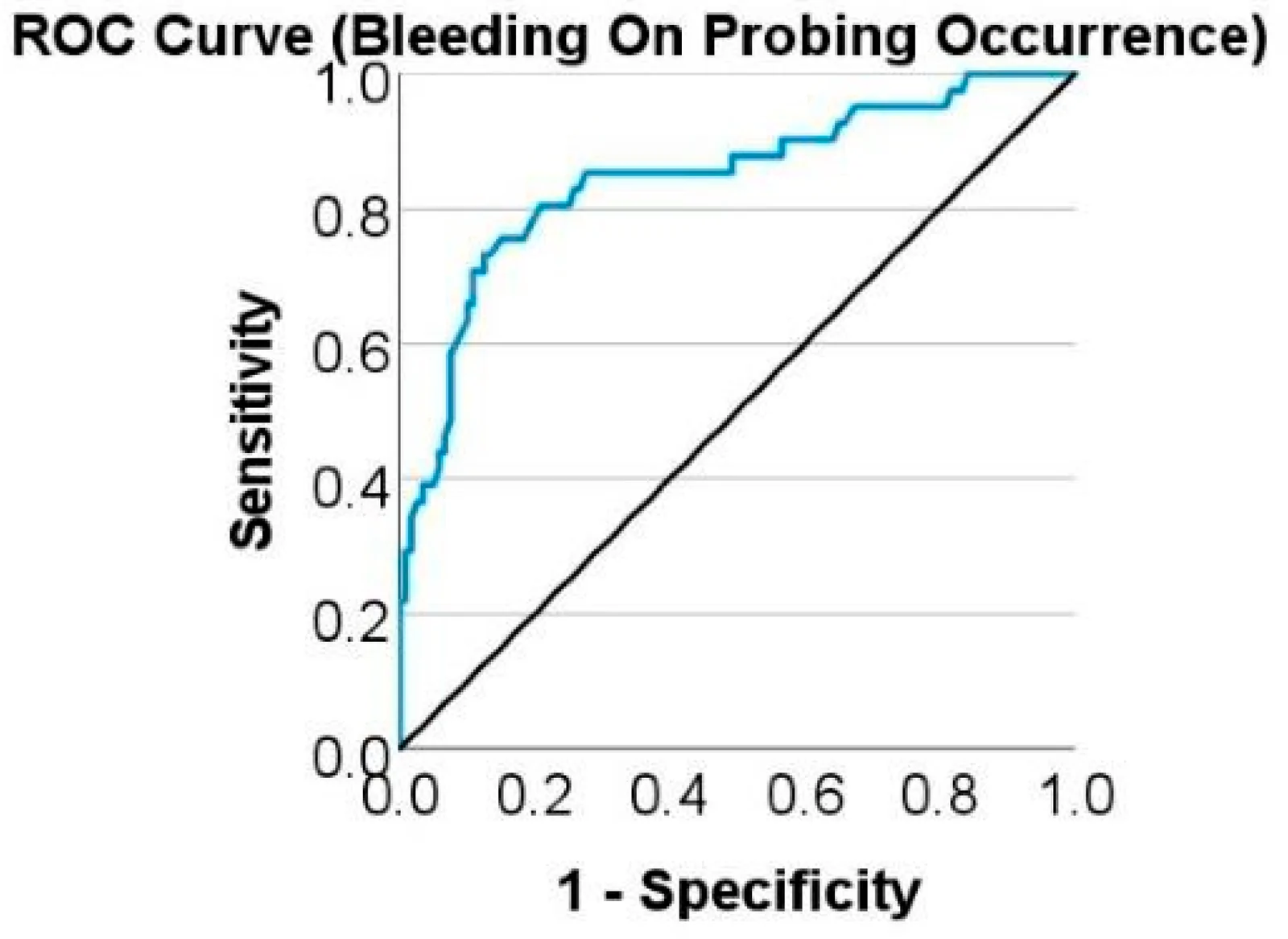
ROC curve demonstrating good discriminative ability for bleeding occurrence.

The ROC curve indicated good discriminatory performance of the regression model, with an AUC of 0.834 (95% CI: 0.758–0.909, *p* < 0.001) (Figure 25).

## 4. Discussion

### 4.1. Principal Findings

The principal finding of this pilot comparative study was that the proposed progressive gingival digital workflow achieved favorable clinical and radiographic outcomes that did not differ significantly from those observed in the control group. No significant differences were identified between the two groups regarding plaque accumulation, bleeding on probing, peri-implant sulcus dimensions, gingival condition, or marginal bone resorption, suggesting that peri-implant stability may be influenced by multiple biological and restorative factors beyond implant design alone. In contrast, plaque accumulation, vestibular sulcus depth, and implant duration emerged as predictors of bleeding on probing, emphasizing the importance of long-term peri-implant maintenance. A further relevant finding is that the proposed protocol enabled clinician-driven abutment customization and progressive soft-tissue conditioning while maintaining favorable clinical and radiographic outcomes, with no statistically significant differences detected between groups for the evaluated parameters.

### 4.2. Clinical Rationale of the Proposed Progressive Gingival Digital Workflow

Evidence suggests that peri-implant soft-tissue maturation continues for several weeks after uncovering [25,34,43]. In this context, initiating chairside abutment customization after early healing, while using provisional restorations for additional tissue conditioning, may support emergence profile development and soft-tissue stability. The importance of careful soft-tissue management as a determinant of healing and long-term stability has also been emphasized in other oral surgical contexts, including regenerative approaches following odontogenic cyst surgery [44]. A distinctive feature of this approach is the direct clinician control over biologically relevant abutment positioning during progressive gingival maturation.

Chairside abutment customization relies on prosthodontic principles familiar from conventional fixed prosthodontics and may represent a clinically feasible alternative to more laboratory-dependent customization approaches. A potential concern associated with chairside modification of prefabricated abutments is the possibility of altering abutment geometry, stress distribution, fatigue resistance, or surface characteristics. Although no abutment- related mechanical complications were observed during the present follow-up period, the study was not designed to evaluate the mechanical consequences of abutment modification.

Furthermore, excessive surface alteration of transmucosal components could theoretically increase plaque retention if appropriate surface finishing is not maintained. Future laboratory and clinical studies are required to investigate the long-term mechanical and biological implications of chairside abutment customization.

Compared with laboratory-dependent workflows, the present approach may reduce certain laboratory-related waiting phases [1,4,8,35]. One practical aspect of the proposed workflow is that the prefabricated stock abutments used in this study provided sufficient axial height for cement-retained restorations while allowing progressive chairside modification according to peri-implant soft tissue maturation. This approach may reduce the need for extensive adjustment of larger universal abutments and offers flexibility during emergence profile development.

Because customized laboratory abutment fabrication is not required in this workflow, the restorative sequence differs from workflows that rely on laboratory-manufactured individualized abutments. However, no direct measurements of laboratory turnaround time, number of appointments, treatment duration, or cost were performed in the present study.

With our proposed approach, the verification of the abutments is included in the same appointment as the preparation, and it is performed visually (again relying on the clinician’s experience from tooth preparation), as well as with the instrument provided by the device’s software.

Any scanning of abutments can generally involve errors [1,36]. The individualized geometry of the abutments may contribute to improved scan visualization during the digital workflow.

From a clinical workflow perspective, the proposed sequence may facilitate treatment organization and progressive soft-tissue management. After scanning, the provisional restoration can be delivered as early as the next day (according to the agreement with the laboratory). The provisional could even be left in place for two weeks for functional testing. Practically, two weeks are usually enough. The final scan is therefore performed with healed gingiva, and the definitive restoration may be delivered after soft-tissue maturation has stabilized. It is noteworthy that during these weeks, the peri-implant gingiva is in contact with titanium abutments rather than PMMA resin. According to the literature, titanium provides a favorable gingival interface, similar to zirconia, which supports healing [45]. The shaping of the cervical margins in the anterior area is performed during the final scan appointment, after which the zirconia crowns—which have been associated with favorable soft-tissue responses—are placed and establish the subgingival interface with the gingiva [45].

The use of the same prosthetic abutments throughout treatment may represent a practical advantage by eliminating the need for additional prosthetic components. However, the economic impact of this approach was not directly evaluated in the present study.

### 4.3. Comparison with the Existing Literature

The established tissue-level protocol was selected as a clinical reference because tissue-level implants have been widely used for many years and their transmucosal design has historically been associated with simplified peri-implant soft tissue management and stable tissue adaptation. Since the proposed progressive gingival digital workflow was specifically developed to support soft tissue conditioning and emergence profile development around bone-level implants, comparison with an established tissue-level restorative concept was considered clinically relevant. The objective was not to compare implant designs in isolation, but rather to evaluate two integrated treatment concepts used in routine clinical practice.

Tissue-level implants are characterized by a transmucosal collar that positions the implant–abutment junction away from the crestal bone and have traditionally been associated with simplified prosthetic procedures and favorable soft-tissue adaptation [30]. Bone-level implants, in contrast, are placed at the level of the alveolar crest and provide greater prosthetic flexibility for emergence profile development and customization of peri-implant soft tissues, particularly in esthetically demanding situations [46].

In contrast, plaque accumulation emerged in the multivariable model as an independent predictor of bleeding on probing, reinforcing the biological relevance of biofilm control for peri-implant tissue stability. This value can be compared with percentages reported in the literature, such as in a comparative study between screw-retained and cement-retained crowns, which showed that bleeding on probing was significantly more frequent in cement-retained implant crowns, reaching 37% of the evaluated implants, while the screw-retained crowns showed a percentage of 21.84% [2]. Our bleeding values were closer to those reported for screw-retained restorations in that study, suggesting acceptable peri-implant hygiene conditions in the present cohort [18,47,48]. The present investigation focused on cement-retained restorations because the proposed treatment concept was developed around progressive abutment customization and emergence profile management. Although screw-retained restorations are often preferred because of their retrievability and elimination of cement-related complications, current evidence does not consistently support the superiority of one retention modality over the other. Therefore, the findings of the present study should be interpreted within the specific context of cement-retained implant-supported restorations.

When comparing the literature from the past 10 years, studies have shown small differences between bone-level and tissue-level implants. Pera et al. reported, in cases of full-arch prosthetic rehabilitation with immediate loading, a slightly higher survival rate for tissue-level implants one year after surgery [49]: a 100% survival rate for tissue-level, while bone-level implants had a 97.37% survival rate. Also, the study reported a total marginal bone loss of 1.324 ± 0.64 mm for bone-level implants, while a total of 1.194 ± 0.30 mm for tissue-level implants [49]. These values are within the range reported in the present study.

In a cross-sectional study comparing the two types of implants, it was observed that, over time, tissue-level implants showed higher percentages of peri-implant mucositis, whereas bone-level implants showed higher percentages of peri-implantitis [50]. In our study, gingival conditions were predominantly normal in both groups, with no statistically significant differences observed between them.

### 4.4. Predictors of Bleeding and Clinical Implications

Beyond comparisons between implant designs, the multivariable regression model suggested that plaque accumulation, vestibular sulcus depth, and implant duration may influence bleeding on probing more strongly than implant macrodesign itself. In particular, the association between increasing plaque levels and bleeding reinforces the established biological relevance of biofilm control in peri-implant tissue stability. Likewise, the association with vestibular sulcus depth may indicate that local soft-tissue morphology and cleansability can influence inflammatory susceptibility. The additional effect of implant duration may reflect the cumulative influence of long-term functional and hygienic factors. Taken together, these findings support the concept that maintenance-related biological parameters may play a greater role in peri-implant tissue behavior than the bone-level or tissue-level implant configuration alone.

### 4.5. Limitations

This study has several limitations that should be acknowledged. First, its retrospective pilot design and modest sample size limit the generalizability of the findings. Second, analyses were performed primarily at the implant level, and the inclusion of multiple implants in some patients may have introduced clustering effects that were not fully accounted for statistically. In addition, the two study groups were restored using implant systems from different manufacturers. Consequently, differences in implant macrodesign, implant–abutment connection, surface characteristics, surgical instrumentation, and manufacturing protocols may have influenced the observed biological and radiographic outcomes independently of the restorative workflow. Therefore, the individual contribution of the prosthetic protocol cannot be isolated from implant-related factors. Furthermore, no formal a priori sample size calculation was performed, and post hoc power analyses suggested limited power for some outcomes. Consequently, the absence of statistically significant differences should not be interpreted as evidence of equivalence between the two treatment protocols.

Third, the comparison involved two implant systems with different macrodesign characteristics, which may represent a potential confounding factor independent of the restorative workflow itself. In addition, patient satisfaction was assessed qualitatively, and no dedicated patient-reported outcome instrument was used. Finally, although the proposed workflow showed promising clinical performance, longer prospective studies with larger cohorts and standardized maintenance-related indices are needed to validate these findings. Because treatment allocation was based on implant system selection and clinical preference rather than randomization, the possibility of selection bias cannot be excluded. Consequently, unmeasured baseline differences between groups may have influenced the observed outcomes.

In addition, implant location (anterior versus posterior region) was not analyzed as a separate study variable; therefore, potential site-specific influences on the observed outcomes could not be assessed.

The potential influence of abutment angulation on biomechanical load distribution was not specifically evaluated. Since abutment angulation was not recorded as a separate study variable, its contribution to the observed clinical outcomes cannot be determined. The multivariable models, particularly for bone resorption, should be interpreted cautiously due to the limited number of events. Because several patients contributed more than one implant, implant-level observations were not fully independent. However, supplementary GEE analyses accounting for patient-level clustering produced results consistent with the primary analyses.

The bone resorption model was based on a limited number of outcome events relative to the number of predictors and should therefore be interpreted as exploratory. Despite these limitations, the present findings support the feasibility of a biologically driven clinician-centered digital workflow as a potentially useful alternative for implant-supported prosthetic rehabilitation.

## 5. Conclusions

The proposed progressive gingival digital workflow enabled clinician-driven prosthetic abutment customization integrated with progressive soft-tissue conditioning and was associated with favorable clinical and radiographic outcomes over a 2–4-year follow-up period. Within the limitations of this retrospective pilot study, no statistically significant differences were observed between the study groups, suggesting that biologically oriented restorative management may contribute to peri-implant stability alongside established biological and prosthetic factors.

The protocol may represent a clinically feasible alternative that supports emergence profile development within a clinician-controlled restorative workflow. Furthermore, the observed influence of maintenance-related factors, particularly plaque accumulation and peri-implant soft tissue conditions, underscores the importance of biological monitoring alongside prosthetic design.

Because the study was exploratory in nature and not designed as an equivalence trial, the absence of statistically significant differences between groups should not be interpreted as evidence of equivalence between the two treatment concepts. Further prospective studies with larger cohorts, longer follow-up periods, and appropriately powered study designs are required to validate the proposed workflow and clarify its long-term clinical performance.

## Figures and Tables

**Table 1:** Clinical stages of the proposed progressive gingival digital workflow.

Stage	Procedure	Primary Objective
1.	Implant uncovering	Initial soft-tissue conditioning
2.	Chairside abutment customization	Abutment parallelization and emergence profile development
3.	Provisional PMMA restoration	Functional and progressive soft-tissue conditioning
4.	Final digital scan	Emergence profile capture and refinement
5.	Definitive zirconia restoration delivery	Definitive prosthetic rehabilitation

**Table 2:** Baseline demographic and clinical characteristics of the study groups.

Variable	Category	Control (*n* = 74)	Experimental (*n* = 87)	*p*-Value
Age (years)	Mean ± SD	60.84 ± 12.08	60.34 ± 11.89	
	Median (range)	63 (34–82)	61 (33–82)	0.996
Sex, *n* (%)	Male	34 (45.95%)	46 (52.87%)	
	Female	40 (54.05%)	41 (47.13%)	0.381
Gingival biotype, *n* (%)	Thin	58 (78.38%)	62 (71.26%)	
	Thick	16 (21.62%)	25 (28.74%)	0.302
Gingival condition, *n* (%)	Healthy	71 (95.95%)	83 (95.40%)	
	Inflamed	2 (2.70%)	4 (4.60%)	
	Recession	1 (1.35%)	0	0.458
Implant position, *n* (%)	11–18	19 (25.68%)	29 (33.33%)	
	21–28	27 (36.49%)	21 (24.14%)	
	31–38	15 (20.27%)	18 (20.69%)	
	41–48	13 (17.57%)	19 (21.84%)	0.343
Implant age (months)	Mean ± SD	36.84 ± 8.32	35.82 ± 7.43	
	Median (range)	36 (24–50)	32 (24–48)	0.333

Data are presented as mean ± standard deviation (SD), median (range), or number (%), as appropriate. Percentages were calculated within each study group.

**Table 3:** Prosthetic and clinical complications recorded during the follow-up period.

Complication	Control (*n* = 79)	Experimental (*n* = 82)
Prosthetic complications		
Provisional restoration fracture	1	0
Decementation of the definitive restoration	0	1
Loss of proximal contact	0	1
Clinical/functional events		
Phonation adaptation difficulties	0	1
Pain associated with prosthetic procedures	0	1

## Data Availability

The original contributions presented in this study are included in the article. Further inquiries can be directed to the corresponding authors.

## Abbreviations

The following abbreviations are used in this manuscript:

AUC	Area under the curve
BOP	Bleeding on probing
CAD/CAM	Computer-aided design/computer-aided manufacturing
CI	Confidence interval
L-PRF	Leukocyte- and platelet-rich fibrin
OR	Odds ratio
PMMA	Polymethyl methacrylate
PTFE	Polytetrafluoroethylene
ROC	Receiver operating characteristic
SD	Standard deviation
SLA	Sandblasted, large-grit, acid-etched
